# Superparamagnetic Spinel-Ferrite Nano-Adsorbents Adapted for Hg^2+^, Dy^3+^, Tb^3+^ Removal/Recycling: Synthesis, Characterization, and Assessment of Toxicity

**DOI:** 10.3390/ijms241210072

**Published:** 2023-06-13

**Authors:** A. F. P. Allwin Mabes Raj, Maja Bauman, Nena Dimitrušev, Lamiaa M. A. Ali, Mélanie Onofre, Magali Gary-Bobo, Jean-Olivier Durand, Aleksandra Lobnik, Aljoša Košak

**Affiliations:** 1 Jožef Stefan International Postgraduate School, Jamova 39, 1000 Ljubljana, Slovenia; allwinamc10@gmail.com; 2 Department of Environmental Science, Jožef Stefan Institute, Jamova 39, 1000 Ljubljana, Slovenia; 3 Institute for Environmental Protection and Sensors (IOS) Ltd., Beloruska 7, 2000 Maribor, Slovenia; 4 Faculty for Mechanical Engineering, University of Maribor, Smetanova 17, 2000 Maribor, Slovenia; 5 IBMM, Univ Montpellier, CNRS, ENSCM, 34293 Montpellier, France; 6 ICGM, Univ Montpellier, CNRS, ENSCM, 34293 Montpellier, France

**Keywords:** nanomaterials, iron oxides, maghemite, cobalt ferrite, adsorption, transition metals, cytotoxicity, terbium, dysprosium, mercury

## Abstract

In the present work, superparamagnetic adsorbents based on 3-aminopropyltrimethoxy silane (APTMS)-coated maghemite (γFe_2_O_3_@SiO_2_–NH_2_) and cobalt ferrite (CoFe_2_O_4_@SiO_2_–NH_2_) nanoparticles were prepared and characterized using transmission-electron microscopy (TEM/HRTEM/EDXS), Fourier-transform infrared spectroscopy (FTIR), specific surface-area measurements (BET), zeta potential (ζ) measurements, thermogravimetric analysis (TGA), and magnetometry (VSM). The adsorption of Dy^3+^, Tb^3+^, and Hg^2+^ ions onto adsorbent surfaces in model salt solutions was tested. The adsorption was evaluated in terms of adsorption efficiency (%), adsorption capacity (mg/g), and desorption efficiency (%) based on the results of inductively coupled plasma optical emission spectrometry (ICP-OES). Both adsorbents, γFe_2_O_3_@SiO_2_–NH_2_ and CoFe_2_O_4_@SiO_2_–NH_2_, showed high adsorption efficiency toward Dy^3+^, Tb^3+^, and Hg^2+^ ions, ranging from 83% to 98%, while the adsorption capacity reached the following values of Dy^3+^, Tb^3+^, and Hg^2+^, in descending order: Tb (4.7 mg/g) > Dy (4.0 mg/g) > Hg (2.1 mg/g) for γFe_2_O_3_@SiO_2_–NH_2_; and Tb (6.2 mg/g) > Dy (4.7 mg/g) > Hg (1.2 mg/g) for CoFe_2_O_4_@SiO_2_–NH_2_. The results of the desorption with 100% of the desorbed Dy^3+^, Tb^3+^, and Hg^2+^ ions in an acidic medium indicated the reusability of both adsorbents. A cytotoxicity assessment of the adsorbents on human-skeletal-muscle derived cells (SKMDCs), human fibroblasts, murine macrophage cells (RAW264.7), and human-umbilical-vein endothelial cells (HUVECs) was conducted. The survival, mortality, and hatching percentages of zebrafish embryos were monitored. All the nanoparticles showed no toxicity in the zebrafish embryos until 96 hpf, even at a high concentration of 500 mg/L.

## 1. Introduction

Transition metals (TM) and internal transition metals (ITM), often referred to as d- and f-block elements, respectively, are key raw materials for the European economy, forming a strong industrial base that produces a wide range of products and applications used in everyday life and modern technologies. Many of these metals are considered to be highly toxic and have negative environmental and human health effects due to anthropogenic factors (e.g., mercury, lead, chromium, etc.). Furthermore, both groups also include strategic metals, such as rare-earth metals (e.g., dysprosium, terbium, samarium, neodymium, etc.), which are in increasing demand and subject to supply risks. Therefore, reliable and unhindered access to these raw materials is a growing concern in the EU and globally [1,2,3,4,5].

Of these metals, mercury (Hg) is particularly noteworthy. It can be found in many common devices, from thermometers, barometers, thermostats, and pressure gauges to fluorescent lamps, etc., and it is considered to be the most toxic heavy metal in the environment, particularly due to its high bioaccumulation and biomagnification capacity [6]. This is because spilled elemental mercury (Hg^0^) is converted by microbial processes in the environment, particularly in water, into an organic form called methylmercury (MeHg), which is the most toxic form of mercury. Methylmercury is then transferred to fish and other wildlife and, eventually, it can be ingested, causing adverse health effects [6,7].

The removal of toxic metals, such as mercury, from the environment plays a significant role in minimizing their environmental and human health effects, while the recycling of essential and precious transition metals, such as platinum, palladium, gold, silver, rhodium, iridium, ruthenium, cobalt, niobium, tungsten, etc., and rare-earth metals, such as terbium, dysprosium, neodymium, lanthanum, samarium, cerium, etc., from e-waste and other raw waste materials, is of major importance to increase the availability of secondary resources, as well as improving the knowledge base that provides prerequisites for a circular economy on a larger scale than today [1,6,8,9,10]. Generally, current processing/removal technologies include, but are not limited to, hydrometallurgy (solvent extraction, ion exchange, precipitation, and crystallization), pyrometallurgy, electrometallurgy (electrorefining, and electrowinning), and aeriometallurgy (supercritical fluid extraction) [11,12].

All these technologies have many disadvantages. Pyrometallurgy is energy-intensive and generates greenhouse gas (GHG) emissions, while hydrometallurgy relies on large volumes of acids and organic solvents, thus generating hazardous wastes [13,14,15]. The primary disadvantage of aeriometallurgy is that the extraction must be operated at the high pressure (1000–5000 psi) required to maintain the solvent in a supercritical state using supercritical CO_2_ [16]. In electrometallurgy, an inert atmosphere is usually required for recycling related to operational and maintenance drawbacks, while the recycling of raw metals can generate a small volume of waste, which is not yet developed, qualified, certified, or accepted. However, the electrometallurgy process also features the drawbacks of huge energy consumption for heating and electrolytic reduction and potential chlorine-gas emission [17,18].

An attractive alternative to these technologies is the solid-phase extraction (SPE) of metal ions from the solution using nanostructured materials as adsorbents, characterized by surface functionality, high surface-to-volume ratio, and/or porosity [19,20]. The use of SPE involves the adsorption of the target-metal ions from the solution onto the adsorbent surface followed by the subsequent recycling of the metals and the regeneration of the adsorbents [21,22]. The advantages of SPE include its low solvent consumption, ease of use, efficient removal of metal ions, even at low concentrations, and automation capabilities.

In the last decade, ferrimagnetic iron-oxide nanoparticles (NPs) have received special attention in the field of adsorbents, as reported in many reviews [23,24,25,26]. Magnetite (Fe_3_O_4_) and maghemite (γFe_2_O_3_) are the main types of ferrimagnetic NP, and so far, they have received considerable attention due to their nontoxicity and biocompatibility [27,28,29], as well as their ability to be easily dispersed and collected using an external magnetic field [30,31,32]. These ferrimagnetic materials, when reduced to particle dimensions smaller than a certain domain, exhibit superparamagnetic behavior, which means that when an external magnetic field is applied, they magnetize to saturation magnetization (*σ*_s_), but when the magnetic field is removed, they no longer exhibit either residual magnetism (*M*_r_) or coercivity (*H*_c_) [33]. Hence, superparamagnetic iron-oxide NPs can be easily guided in the magnetic field [25,27,34,35]. One prominent example is the introduction of cobalt (Co^2+^) ions into an iron-oxide spinel crystal lattice. Cobalt-doped iron oxides, also known as cobalt ferrites (CoFe_2_O_4_), arouse interest in adsorption applications as their magneto-crystalline anisotropy, which affects the magnetization, coercivity, reversal, and relaxation of nanoparticles, can be tuned by the substitution of cobalt for iron [36,37,38,39,40].

Due to the increasing use of iron-oxide NPs as adsorbents in the recycling of strategic transition metals, there is a high likelihood that these NPs may ultimately enter aquatic ecosystems through effluent discharge and leaching during or after recycling activities, thereby affecting the environment and human health. Unfortunately, there is a serious lack of accurate and sufficient information on their toxic effects. Therefore, toxicity assessment has become increasingly important to understand the impact of these NPs on human health and the environment [41].

In many studies, it was found that uncoated NPs usually tend to be more toxic than coated particles; therefore, the surface modification of uncoated NPs can significantly reduce their toxicity [41,42,43,44,45,46,47,48,49]. Other studies revealed significant cytotoxic effects of these NPs, such as inflammation, the formation of apoptotic bodies, impaired mitochondrial function (MTT), the leakage of membrane lactate dehydrogenase (LDH assay), the generation of reactive oxygen species (ROS), increases in the number of micronuclei as indicators of gross chromosomal damage (a measure of genotoxicity), and chromosome condensation [44,48,50,51,52,53,54].

Furthermore, little is known about the toxicity of the metal dopants of iron-oxide NPs such as cobalt (Co^2+^). The potential toxicity of cobalt-doped iron oxide (CoFe_2_O_4_) is therefore the subject of many debates concerning environmental, health, and safety issues, particularly their use in the environment and their effects on human health [28,44,55,56,57,58,59,60,61,62].

One of the simplest ways to modify the surfaces of NPs is to use alkoxysilanes, which are considered among the preferred coating materials due to their chemical stability, biocompatibility [63,64,65], and versatility, to achieve the functionality [65,66] required in the end-use applications of NPs.

To ensure the functionality of iron-oxide NPs, various alkoxysilane ligands can be grafted directly to their surfaces in one step, avoiding an intermediate multistage reaction mechanism. The grafting principle of alkoxysilanes is based on the sol-gel hydrolysis of alkoxide groups in the structures of alkoxysilane precursors, producing silanol groups (Si-OH), which undergo condensation reactions to form siloxane bonds (Si–O–Si) on the surface of the iron oxide, resulting in the formation of a protective silica surface layer (SiO_2_). Many alkoxysilanes may contain various functional groups in their aliphatic chains, such as hydroxyl (–OH), amine (–NH_2_), mercapto (–SH), carboxylic (–COOH), sulphonic (–SO_3_H), phosphonate (–PO(OH)_2_), phosphate (–PO_2_(OH)_2_), etc., which contain electron-donor atoms (O, N, P, S) and allow the formation of relatively strong complexes with the target transition-metal ions to be recycled [31,41,42,45,67,68,69,70,71].

Although many studies report that alkoxysilanes are non-toxic [18,19,20,71,72,73], their toxicity in terms of reactivity, stability, and degradation effects has not yet been thoroughly investigated [74,75,76,77].

In the present work, we attempted to fabricate efficient superparamagnetic adsorbents based on two different spinel-type iron oxides, both maghemite (γFe_2_O_3_) and cobalt ferrite (CoFe_2_O_4_), which were surface-functionalized using a (3-aminopropyl)trimethoxy silane (APTMS) precursor. The functionalized superparamagnetic adsorbents were characterized to test their adsorption efficiency and adsorption capacity towards Dy^3+^, Tb^3+^, and Hg^2+^ ions in aqueous solutions and their desorption efficiency when an acidic medium was used. The assessment of the cytotoxicity of both types of NP with and without an amino-propyl (–(CH_2_)_3_NH_2_) surface coating was conducted on four different types of healthy cell: human-skeletal-muscle-derived cells, human fibroblasts, murine macrophages cells, and human-umbilical-vein endothelial cells. Further, their toxic effects on zebrafish embryos were also evaluated by recording the survival, mortality, and hatching percentages during embryo development.

## 2. Results and Discussion

### 2.1. Synthesis and Characterization of MNPs

**Figure 1:**
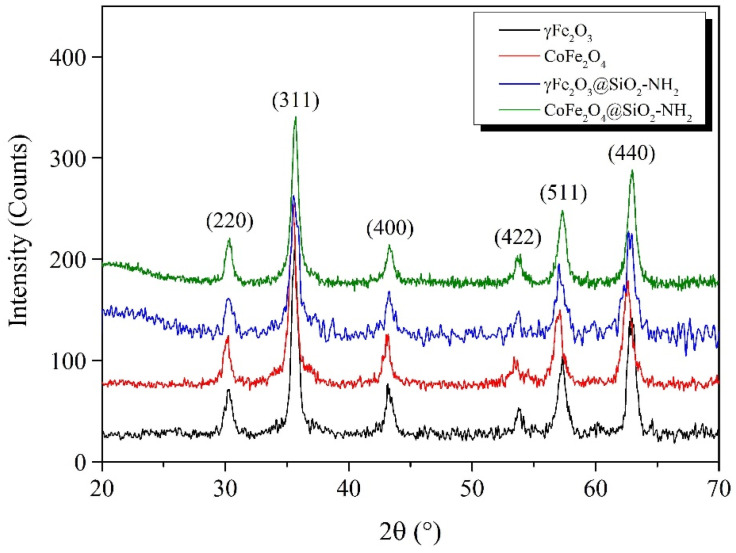
XRD patterns of γFe_2_O_3_ and CoFe_2_O_4_ NPs.

The magnetic γFe_2_O_3_ and CoFe_2_O_4_ NPs prepared by the coprecipitation method were characterized using XRD (Figure 1). All the diffraction peaks of the prepared samples were consistent with the cubic spinel crystal structure (JCPDS Card 39-1346). It can be seen from the XRD pattern that the presence of diffraction lines at 2*θ* of 30.5°, 35.5°, 43.2°, 53.6°, 57.1°, and 62.9° for both samples corresponded to the cubic crystal planes of (220), (311), (400), (422), (511), and (440), respectively. The particle sizes of the γFe_2_O_3_ and CoFe_2_O_4_ were calculated from the broadening of the most intensive diffraction peak corresponding to the (311) crystal plane using the Deby–Scherrer equation [78,79]. The calculated average particle sizes of the γFe_2_O_3_ and CoFe_2_O_4_ were 10.2 nm and 11.5 nm, respectively, and the crystalline-lattice parameters corresponding to the cubic spinel crystal structure obtained based on Bragg’s law were 0.8358 nm and 0.8345 nm, respectively. The presence of broad amorphous diffraction peaks for the functionalized γFe_2_O_3_@SiO_2_–NH_2_ and CoFe_2_O_4_@SiO_2_–NH_2_ NPs, which appeared at a low diffraction angle 2*θ* of 20°, was due to the presence of the amorphous SiO_2_ surface layer, indicating that the crystalline cubic spinel γ-Fe_2_O_3_ and CoFe_2_O_4_ NPs were successfully surface-functionalized with APTMS [80].

The positions of the diffraction peaks for the γFe_2_O_3_@SiO_2_–NH_2_ and CoFe_2_O_4_@SiO_2_-NH_2_ NPs were at the same positions 2*θ* as those of the γFe_2_O_3_ and CoFe_2_O_4_, indicating that the crystalline cubic spinel structures remained unchanged after their functionalization with APTMS.

**Figure 2:**
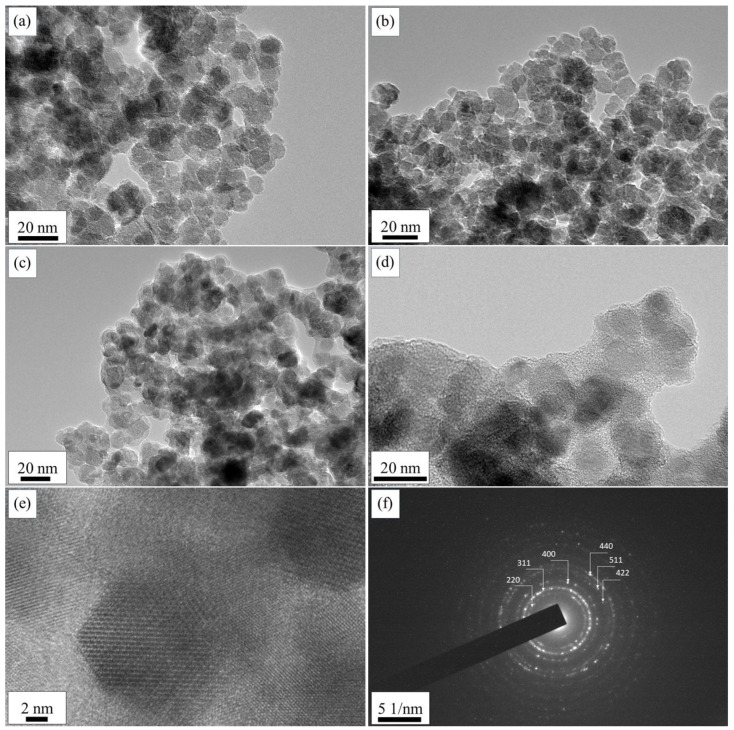
TEM micrographs of (**a**) γFe_2_O_3_, (**b**) CoFe_2_O_4_, (**c**) γFe_2_O_3_@SiO_2_–NH_2_, (**d**) CoFe_2_O_4_@SiO_2_–NH_2_, (**e**) high-resolution image (HRTEM), and (**f**) electron-diffraction pattern of spinel γFe_2_O_3_ and CoFe_2_O_4_ NPs.

The transmission-electron micrographs in Figure 2 represent the morphological properties of the as-prepared γFe_2_O_3_ and CoFe_2_O_4_ NPs and functionalized Fe_2_O_3_@SiO_2_–NH_2_ and CoFe_2_O_4_@SiO_2_–NH_2_ core@shell nanostructures. It can be seen that the obtained γFe_2_O_3_ and CoFe_2_O_4_ NPs were relatively spherical in shape, with average particle sizes of (9.9 ± 0.9) nm and (11.5 ± 1.0) nm, respectively, while the particle-size distributions of the functionalized γFe_2_O_3_@SiO_2_–NH_2_ and CoFe_2_O_4_@SiO_2_–NH_2_ NPs were (14.5 ± 1.1) nm and (17.7 ± 1.2) nm, respectively. The electron-diffraction patterns of the γFe_2_O_3_ and CoFe_2_O_4_ NPs indicated the crystalline nature of the prepared powders, with each of the concentric diffraction rings belonging to the spinel crystal structure.

**Figure 3:**
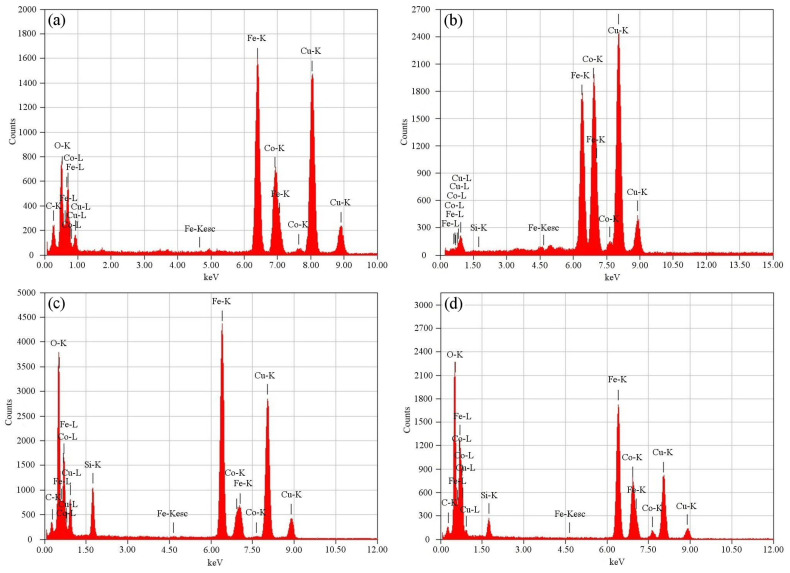
EDXS spectra of (**a**) γFe_2_O_3_, (**b**) CoFe_2_O_4_, (**c**) γFe_2_O_3_@SiO_2_–NH_2_, and (**d**) CoFe_2_O_4_@SiO_2_–NH_2_ NPs.

The EDXS patterns of the γFe_2_O_3_ and CoFe_2_O_4_ NPs in Figure 3a,b confirm the presence of Co, Fe, and O elements, indicating the formation of γFe_2_O_3_ and CoFe_2_O_4_ nanostructures, while on the EDXS spectrum of the γFe_2_O_3_@SiO_2_–NH_2_ and CoFe_2_O_4_@SiO_2_–NH_2_ in Figure 3c,d, respectively, the presence of C, O(N), Co, Fe, and Si confirmed the success of the surface functionalization of the γFe_2_O_3_ and CoFe_2_O_4_ NPs with APTMS and, thus, the formation of the core@shell nanostructures. Small proportions of Cu and C elements belong to the TEM copper-grid supported transparent carbon foil.

The Brunauer–Emmet–Teller (BET) analysis showed specific surface areas of 94.9 m^2^/g for the γFe_2_O_3_ and 62.5 m^2^/g for the CoFe_2_O_4_. According to the BET-specific surface area at a relative pressure of 0.3, average particle sizes (*d*_bet_) of 15.1 nm and 18.5 nm were calculated for the γFe_2_O_3_ and CoFe_2_O_4_, respectively, assuming the sphericity of the NPs using the equation *S_bet_* = 6/(*d*_bet_∙*ρ*), where *ρ* is a theoretical density of 4.9 g/cm^3^ for γFe_2_O_3_ [81] and 5.2 g/cm^3^ for CoFe_2_O_4_ [82]. The average size calculated from the surface area was a little higher than that determined using the XRD, most probably due to the agglomeration of the particles [83]. For the Barrett–Joyner–Halenda (BJH) adsorption, the average pore size was 7.0 nm, with a total pore volume of 0.2335 cm^3^/g, for the γFe_2_O_3_ NPs, and 5.8 nm, with a total pore volume of 0.1275 cm^3^/g, for the CoFe_2_O_4_ NPs. Furthermore, for the BJH desorption, the average pore size was 8.4 nm, with a total pore volume of 0.3152 cm^3^/g, for the γFe_2_O_3_, and 6.1 nm, with a total pore volume of 0.1353 cm^3^/g, for the CoFe_2_O_4_ NPs.

Due to the surface functionalization of the γFe_2_O_3_ and CoFe_2_O_4_ NPs with APTMS, the obtained specific surface area decreased to 40.5 m^2^/g for the γFe_2_O_3_@SiO_2_–NH_2_ and 44.7 m^2^/g for the CoFe_2_O_4_@SiO_2_–NH_2_. It is known that the larger the surface area, the smaller the particle size, and a smaller BET surface area means a larger particle size. According to the specific surface areas, the average particle sizes of the prepared γFe_2_O_3_@SiO_2_–NH_2_ and CoFe_2_O_4_@SiO_2_–NH_2_ samples were calculated as 30.2 nm and 25.8 nm, respectively.

**Figure 4:**
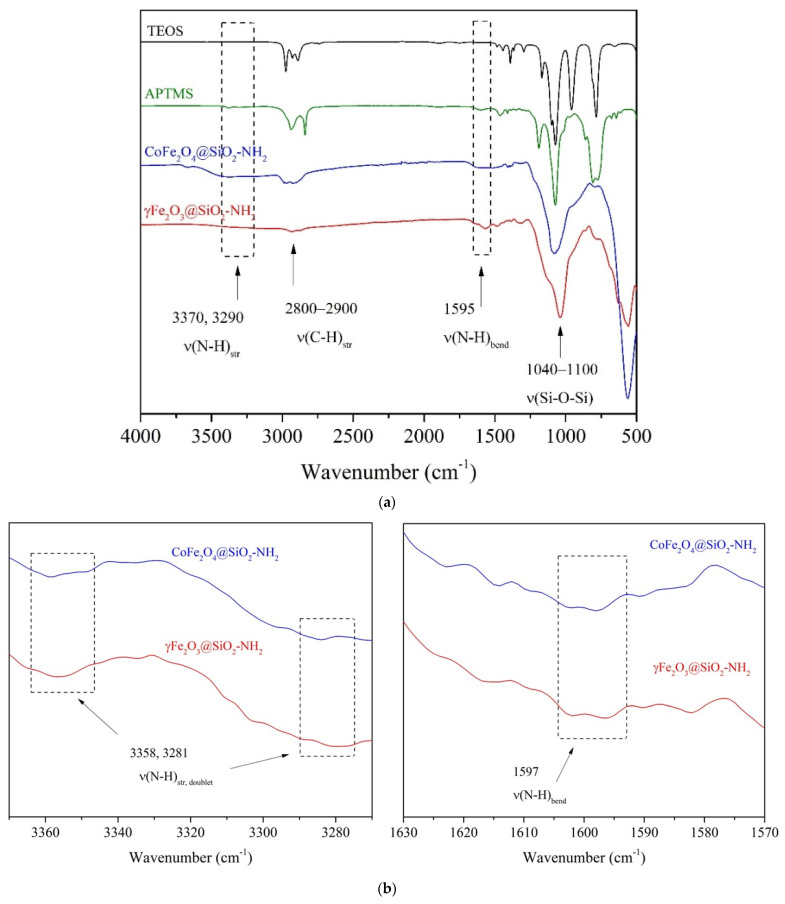
(**a**) FTIR spectra of as-prepared γFe_2_O_3_@SiO_2_–NH_2_ and CoFe_2_O_4_@SiO_2_–NH_2_ NPs and pure alkoxide precursors TEOS and APTMS, and (**b**) enlarged area corresponding to vibrations of amino groups.

A FTIR analysis (Figure 4) was performed to obtain additional information on the coverage of the NPs with the APTMS.

In the FTIR spectra (Figure 4a), the two peaks near 3400 cm^−1^ and 1630 cm^−1^ were assigned to the hydroxyl group OH for all the synthesized NPs. The functionalization process of the γFe_2_O_3_ and CoFe_2_O_4_ NPs with alkoxysilanes was verified by the asymmetric stretching vibrations of the Si-O-Si bonds at 1050 cm^−1^ and the bending of the Si-H bonds at 796 cm^−1^ and at 988 cm^−1^, indicating the formation of silica (SiO_2_) shells.

The presence of amino-propyl groups in the γFe_2_O_3_@SiO_2_–NH_2_ and CoFe_2_O_4_@SiO_2_–NH_2_ samples was confirmed by the peaks at 2934 cm^−1^, 1615 cm^−1^, 1336 cm^−1^, and 781 cm^−1^, which were assigned to the stretching vibrations of the –CH_2_–NH_2_ bonds, the bending of N–H and NH_2_, the wagging and twisting of –CH_2_–NH_2_, and the wagging and twisting of primary amino groups (–NH_2_), respectively. These peaks in the source spectra were not sufficiently visible, but enlarged individual peak areas confirmed their presence (Figure 4b).

**Figure 5:**
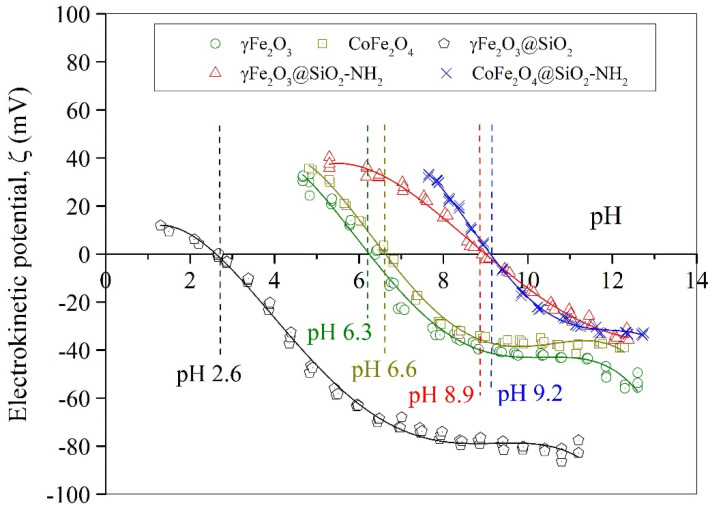
The electrokinetic (ζ) potential of the prepared NPs.

A thermogravimetric analysis (Figure S1) was used to determine the thermal stability and the percentage of amino-propyl ligands grafted onto the surface of the magnetic γFe_2_O_3_ and CoFe_2_O_4_ NPs. According to the literature, the estimation of mass-loss values of 1.1% and 1.3% (not shown in Figure 5) while heating as-prepared magnetic γFe_2_O_3_ and CoFe_2_O_4_ NPs up to 200 °C usually corresponds to the evaporation of physically and chemically absorbed moisture. Further heating of the γFe_2_O_3_ and CoFe_2_O_4_ magnetic NPs up to 900 °C, respectively, resulted in additional mass losses of 2.7% and 3.4%, respectively, which were most likely due to phase and surface changes, the reduction in porosity, and the degradation of the remaining surface species.

The mass losses of the prepared γFe_2_O_3_@SiO_2_–NH_2_ and CoFe_2_O_4_@SiO_2_–NH_2_ samples of 3.1% and 1.8%, respectively (not shown in Figure S1), began at the initial 30 °C mark and continued evolving up to 150 °C. These changes were related to the evaporation of the absorbed moisture from their structures. Further heating of the γFe_2_O_3_@SiO_2_–NH_2_ and CoFe_2_O_4_@SiO_2_–NH_2_ samples up to 900 °C caused more remarkable mass losses of 21.5% and 20.1%, respectively, which corresponded to the decomposition of the SiO_2_ shell and the removal of amino-propyl groups from the NP surfaces, followed by the reduction in the porosity and the cracking of the residual siloxane species, Si–O–Si.

To establish the stability of the prepared γFe_2_O_3_, CoFe_2_O_4,_ γFe_2_O_3_@SiO_2_–NH_2_, and CoFe_2_O_4_@SiO_2_–NH_2_ NPs in an aqueous medium and to determine their surface potential and isoelectric points (IEP), the electrokinetic (ζ) potential as a function of pH media was measured (Figure 5). The pH values of the IEPs for the γFe_2_O_3_ and CoFe_2_O_4_ NPs were about 6.3 and 6.6, respectively, while the silica-coated NPs showed pH dependencies similar to that of pure silica, i.e., at pH 2–3 [69,84].

The values of the ζ-potential for the γFe_2_O_3_ and CoFe_2_O_4_ NPs were higher than +30 mV at pH < 5.2 and lower than −30 mV at pH > 7.9, which means that the γFe_2_O_3_ and CoFe_2_O_4_ NPs were stable in the aqueous media at pH smaller than 5.2 and higher than 7.9. In that pH range, the electrostatic repulsions between the NPs dispersed in an aqueous medium are stronger than the random thermal Brownian motion and, therefore, prevent them from accidental collision and agglomeration and, subsequently, from settling out.

The observed IEP at pH 2.4 for the γFe_2_O_3_@SiO_2_ NPs confirmed that the silica-coating process of the γFe_2_O_3_ NPs was effective, since the charged surface properties were close to those of pure silica (i.e., at pH 2–3) [84]. The silica shell at the maghemite cores caused an increase in their chemical stability at pH values > 4.1, where the surface potential was lower than −30 mV, thus rendering their performance suitable for environmental applications. Moreover, the silica coating prevented the dissolution of the γFe_2_O_3_ and CoFe_2_O_4_ NPs and, thus the leaching of potentially toxic Co^2+^ ions from the spinel crystalline structure and the Fe^2+^/Fe^3+^ oxidation, which otherwise occurs in an acidic medium at values of pH < 3.

It can be seen that an APTMS precursor may be employed to functionalize γFe_2_O_3_ and CoFe_2_O_4_ NPs to form functional γFe_2_O_3_@SiO_2_–NH_2_ and CoFe_2_O_4_@SiO_2_–NH_2_ core@shell nanostructures. The presence of an amine layer on the surface of the γFe_2_O_3_@SiO_2_–NH_2_ and CoFe_2_O_4_@SiO_2_–NH_2_ NPs makes them positive in a broad range of pH due to the protonation and deprotonation of the amine groups, which depend on the solution’s pH values.

The zeta (ζ) potential measurement of the γFe_2_O_3_@SiO_2_–NH_2_ and CoFe_2_O_4_@SiO_2_–NH_2_ NPs in an aqueous solution showed an isoelectric point at about pH 9.0 and stability of the NPs at pH < 6.6 for the γFe_2_O_3_@SiO_2_-NH_2_ and at pH < 7.9 for the CoFe_2_O_4_@SiO_2_–NH_2_, where the ζ–potential was higher than +30 mV, and at pH > 10.6, where the ζ–potential was lower than −30 mV.

We used pH potentiometric titrations for the determination of the total charge of the aqueous colloidal dispersions of the γFe_2_O_3_@SiO_2_–NH_2_ and CoFe_2_O_4_@SiO_2_–NH_2_ NPs.

The results of the potentiometric titration isotherms for the γFe_2_O_3_@SiO_2_–NH_2_ and CoFe_2_O_4_@SiO_2_–NH_2_ NPs are presented in Figure S2. The data exhibited protonation and deprotonation progress for both samples at an alkaline pH of around 10, exhibiting a pKa value of 10.1 for the γFe_2_O_3_@SiO_2_–NH_2_ with a maximum charge of 0.0485 mmol/g and a pKa value of 9.9 for the CoFe_2_O_4_@SiO_2_–NH_2_ with a maximum charge of 0.0155 mmol/g. This can be attributed to the contribution of the total primary amine groups, which is in agreement with data published elsewhere [85,86], and indicates the successful surface functionalization of the MNPs with APTMS.

**Figure 6:**
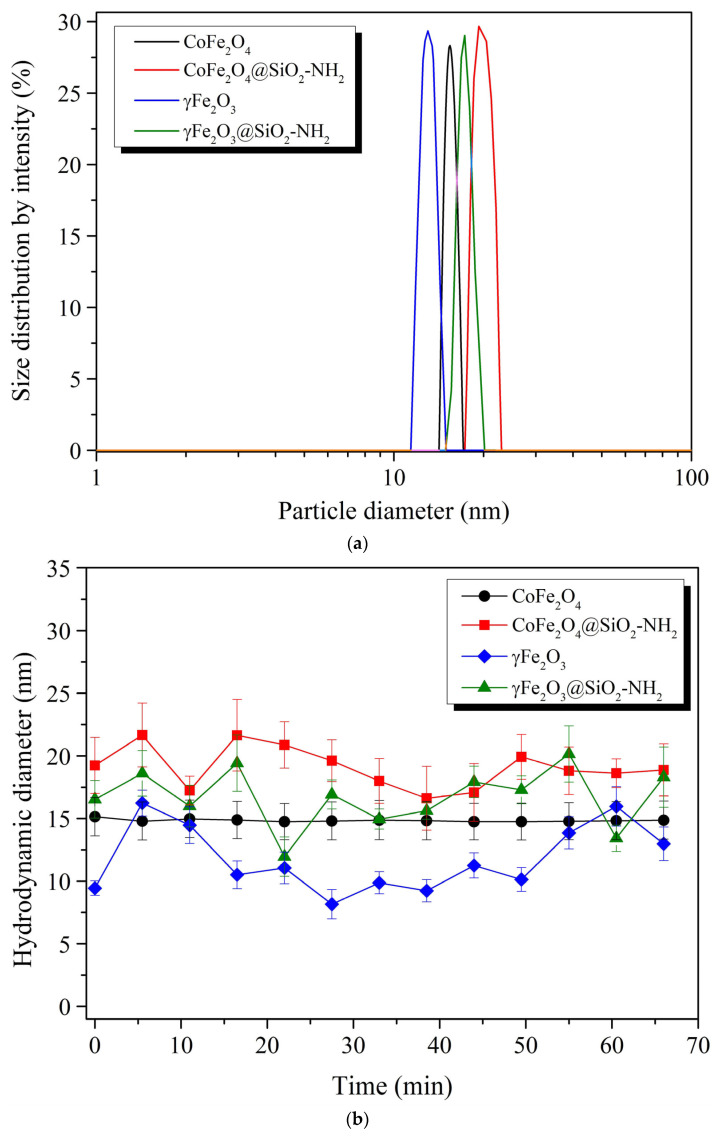
(**a**) Hydrodynamic particle-size distribution for γFe_2_O_3_, CoFe_2_O_4_, γFe_2_O_3_@SiO_2_–NH_2_, and CoFe_2_O_4_@SiO_2_–NH_2_ NPs, and (**b**) the time-dependent hydrodynamic diameters of uncoated γFe_2_O_3_ and CoFe_2_O_4_, and coated γFe_2_O_3_@SiO_2_-NH_2_ and CoFe_2_O_4_@SiO_2_-NH_2_ NPs.

Figure 6a shows the hydrodynamic size distribution of the aqueous colloidal γFe_2_O_3_, CoFe_2_O_4_, γFe_2_O_3_@SiO_2_–NH_2_, and CoFe_2_O_4_@SiO_2_–NH_2_ NPs at 21 °C, according to the intensity-distribution pattern, showing a narrow distribution with homogeneous sizes. The γFe_2_O_3_ and CoFe_2_O_4_ NPs, which were an average diameters of 9.9 nm and 11.5 nm by the TEM, in fact exhibited slightly larger hydrodynamic sizes of approximately 11.7 nm and approximately 14.5 nm, respectively (Figure 6a). On the other hand, after the surface functionalization with the APTMS, the γFe_2_O_3_@SiO_2_–NH_2_ and CoFe_2_O_4_@SiO_2_–NH_2_ NPs showed larger hydrodynamic sizes, of about 16.6 nm and about 19.1 nm, respectively (Figure 6a), compared to the previous primary particle sizes of the same nanoparticles observed by TEM.

It is worth noting that these hydrodynamic sizes were maintained over the applied time range of 1 h (Figure 6a), indicating that both the uncoated γFe_2_O_3_ and CoFe_2_O_4_ and the surface-functionalized γFe_2_O_3_@SiO_2_–NH_2_ and CoFe_2_O_4_@SiO_2_–NH_2_ NPs retained colloidal stability.

The time-dependent hydrodynamic diameters of uncoated γFe_2_O_3_ and CoFe_2_O_4_, and coated γFe_2_O_3_@SiO_2_–NH_2_ and CoFe_2_O_4_@SiO_2_–NH_2_ NPs, are shown in Figure 6b.

Figure 6b shows the samples showed a trend towards colloidal stability. The hydrodynamic diameter of the colloidal CoFe_2_O_4_ NPs did not change significantly with time. The average hydrodynamic size of the colloidal CoFe_2_O_4_ NPs was maintained at approximately (11.7 ± 1.1) nm over the entire time range. The colloidal γFe_2_O_3_ NPs also had a similar behavioral pattern in terms of hydrodynamic size, with a slightly larger fluctuation in the values around the average diameter of the NPs of (14.8 ± 1.5) nm. Although the overall maximum average hydrodynamic sizes of the γFe_2_O_3_ and CoFe_2_O_4_ NPs increased with respect to the particle-size values estimated from the XRD and TEM images, no agglomeration or aggregation of NPs was observed.

After the surface functionalization of the γFe_2_O_3_ and CoFe_2_O_4_ NPs by the APTMS, a fluctuation and an increase in the hydrodynamic diameters of the γFe_2_O_3_@SiO_2_–NH_2_ and CoFe_2_O_4_@SiO_2_–NH_2_ NPs by about 42% and 30%, respectively, were observed in relation to the uncoated γFe_2_O_3_ and CoFe_2_O_4_ NPs. The final hydrodynamic sizes of the γFe_2_O_3_@SiO_2_–NH_2_ and CoFe_2_O_4_@SiO_2_–NH_2_ NPs were 16.6 ± 1.5 nm and 19.1 ± 2.0 nm, respectively, compared to the particle-size values estimated from the XRD and TEM images. Despite the fluctuation and increase in the total maximum hydrodynamic sizes of the γFe_2_O_3_@SiO_2_–NH_2_ and CoFe_2_O_4_@SiO_2_–NH_2_ NPs, no agglomeration or aggregation of NPs was observed. The samples showed a trend toward colloidal stability.

It is obvious that the average particle sizes measured by the DLS technique were slightly larger than the average particle sizes estimated on the basis of the XRD and TEM. It is known that the hydrodynamic sizes of particles dispersed in liquids are usually larger than the primary particle sizes, as reported by many other studies [87,88,89,90]. The hydrodynamic sizes of particles measured by DLS depend on many factors, particularly the concentration of the dispersion, temperature, pH, etc., due to which dispersed nanoparticles may tend to aggregate; therefore, the measured hydrodynamic diameters in such cases are usually much larger than the actual sizes [88,90].

**Figure 7:**
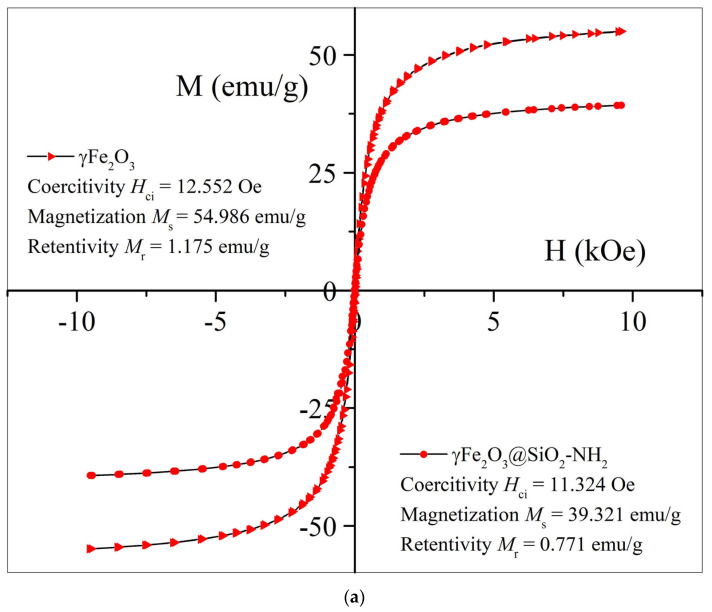

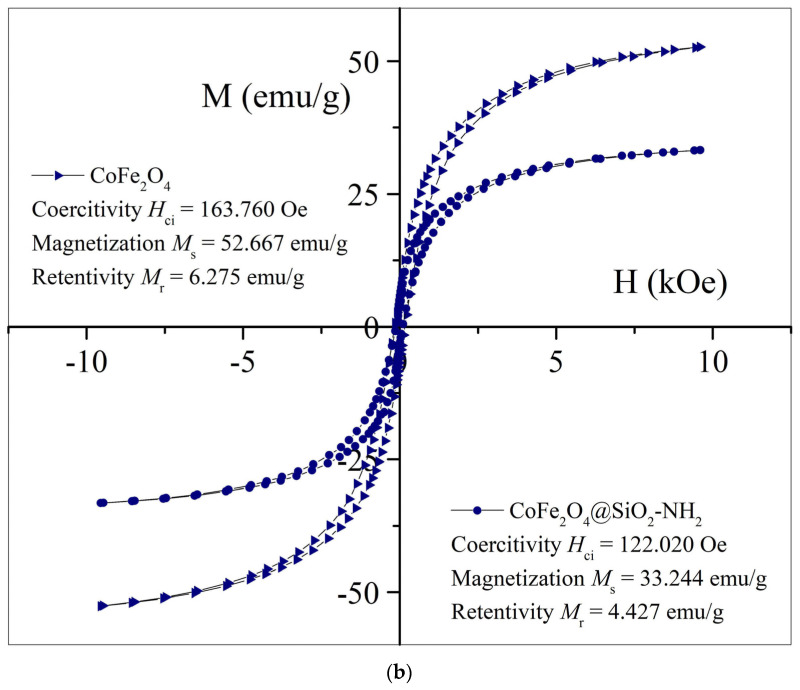
*M-H* curves of the prepared samples of (**a**) γ-Fe_2_O_3_ and γ-Fe_2_O_3_@SiO_2_–NH_2_ NPs, and (**b**) CoFe_2_O_4_ and CoFe_2_O_4_@SiO_2_–NH_2_ NPs.

Figure 7 shows the comparison of the magnetic properties of the uncoated γFe_2_O_3_ and CoFe_2_O_4_ NPs and the functionalized γFe_2_O_3_@SiO_2_–NH_2_ and CoFe_2_O_4_@SiO_2_–NH_2_ NPs, which were carried out using VSM analysis.

At the maximum magnetic field (*H*) strength in the magnetization phase, the specific mass magnetization (*M*_s_) of the samples γFe_2_O_3_ and CoFe_2_O_4_ reached values of 54.98 emu/g and 52.67 emu/g, respectively. The remanent magnetization (*M*_r_) and coercivity (*H*_ci_) values for the samples γFe_2_O_3_ and CoFe_2_O_4_ can be determined from the shape of the hysteresis curves in the vicinity of the zero-magnetic-field strength. We determined that the remanent magnetization and coercivity of the γFe_2_O_3_ sample were 1.17 emu/g and 12.55 Oe, respectively, and that the remanent magnetization and coercivity of the CoFe_2_O_4_ sample were 6.27 emu/g and 163.76 Oe, respectively.

As can be seen, the γFe_2_O_3_ NPs showed very low coercivity due to the small particle sizes. In this case, the γFe_2_O_3_ NPs exhibited a superparamagnetic character; in other words, they were monodomain. In contrast, when the iron in the spinel crystal structure of the maghemite (γFe_2_O_3_) replaced the cobalt (CoFe_2_O_4_), the coercivity was non-zero, and the samples did not show superparamagnetic behavior.

A comparison of the magnetic characteristics of these two samples showed that with the integration of the cobalt into the γFe_2_O_3_ spinel crystal structure, the coercivity increased by thirteen times, and the remanent magnetization increased by almost five times, while the specific mass magnetization did not change significantly.

The increase in coercivity was the cause of the increase in the magnetocrystalline anisotropy due to the cobalt substitution [39]. Magnetocrystalline anisotropy is a key factor that determines the superparamagnetic behavior of nanocrystalline particles and serves as an energy barrier to block spin relaxation, which changes the magnetic state from ferrimagnetic to superparamagnetic [37,38].

The average particle sizes of samples γFe_2_O_3_ and CoFe_2_O_4_ were approximately similar (10.2 nm for γFe_2_O_3_ and 11.5 nm for CoFe_2_O_4_), so the loss of superparamagnetic properties when replacing the iron with the cobalt in the γFe_2_O_3_ spinel crystal structure may have been due to an increase in the magnetocrystalline anisotropy of the CoFe_2_O_4_ [37].

In the magnetization step, under the maximum magnetic field strength (*H_ci_*), the specific mass magnetization (*M*_s_) of the synthesized γFe_2_O_3_@SiO_2_–NH_2_ decreased from 54.98 emu/g for the γFe_2_O_3_ NPs to 39.32 emu/g, and from 52.67 emu/g for the CoFe_2_O_4_ NPs to 33.24 emu/g for the CoFe_2_O_4_@SiO_2_–NH_2_ NPs. This was due to the presence of a non-magnetic SiO_2_-NH_2_ coating on the surfaces of the γFe_2_O_3_ and CoFe_2_O_4_ cores, which, due to its diamagnetic quality, contributed to the reduction in the net-specific magnetization of the γFe_2_O_3_@SiO_2_–NH_2_ and CoFe_2_O_4_@SiO_2_–NH_2_ NPs. After the surface functionalization of the samples γFe_2_O_3_ and CoFe_2_O_4_, a pronounced decrease in the remanence (*M*_r_) and coercivity (*H*_ci_) of the samples was noticed because of the increase in the average particle size at the expense of the SiO_2_-NH_2_ surface coating. The remanence (*M*_r_) thus decreased from 1.17 emu/g for the sample γFe_2_O_3_ to 0.77 emu/g for the sample γFe_2_O_3_@SiO_2_–NH_2_, and from 6.27 emu/g for the sample CoFe_2_O_4_ to 4.43 emu/g for the sample CoFe_2_O_4_@SiO_2_-NH_2_, while the coercivity of the sample γFe_2_O_3_@SiO_2_–NH_2_ decreased to 11.32 Oe from 12.55 Oe for the γFe_2_O_3_, and for the sample CoFe_2_O_4_@SiO_2_-NH_2_ m it decreased to 122.02 Oe from 163.76 Oe for the CoFe_2_O_4_ NPs. The decrease in saturation magnetization (*M*_s_), coercivity (*H*_ci_), and remanent magnetization (*M*_r_) in the γFe_2_O_3_@SiO_2_–NH_2_ and CoFe_2_O_4_@SiO_2_–NH_2_ NPs compared to the γFe_2_O_3_ and CoFe_2_O_4_ NPs was expected due to the larger NP sizes, corresponding to the SiO_2_ shell and the functionalization with amino (–NH_2_) groups.

### 2.2. Adsorption and Desorption Tests

**Figure 8:**
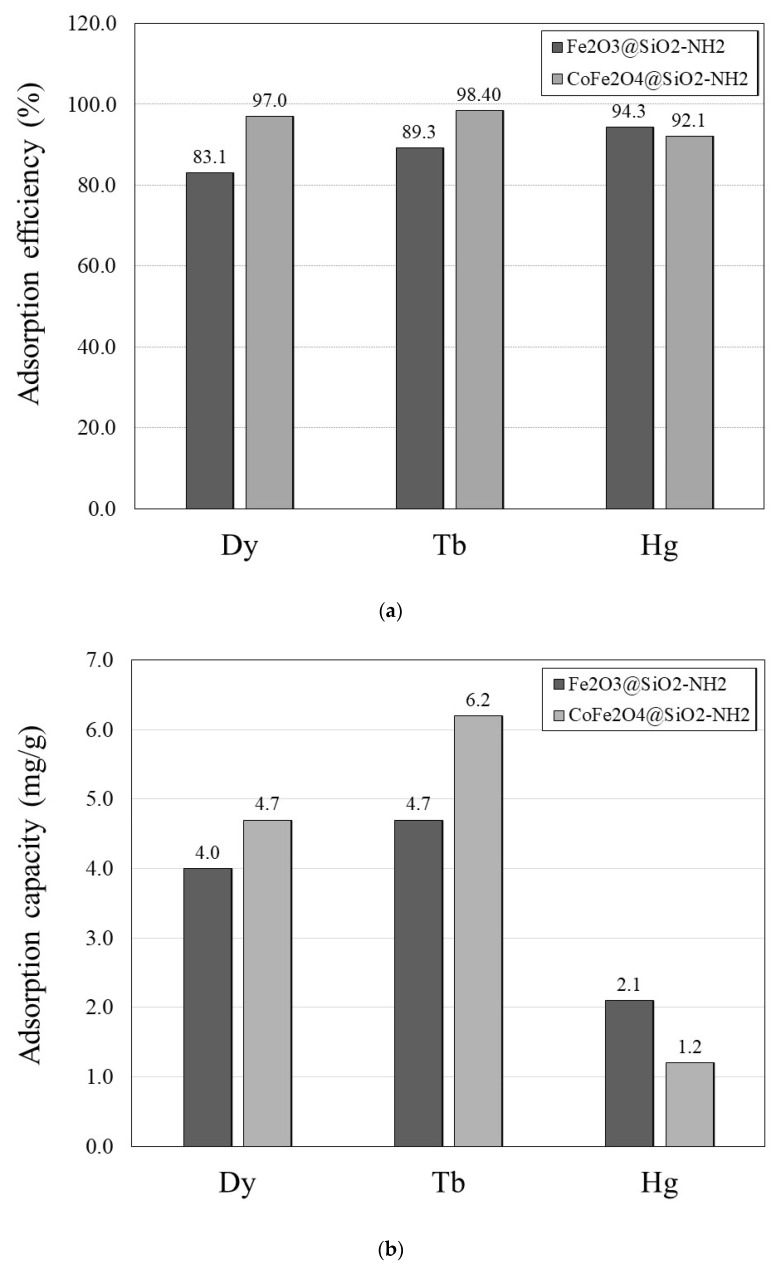
(**a**) Adsorption efficiency (%) and (**b**) adsorption capacity (mg/g) of γFe_2_O_3_@SiO_2_–NH_2_ and CoFe_2_O_4_@SiO_2_–NH_2_ NPs toward Dy^3+^, Tb^3+^, and Hg^2+^ ions.

To evaluate the performances and basic adsorption affinity of the γ-Fe_2_O_3_@SiO_2_–NH_2_ and CoFe_2_O_4_@SiO_2_–NH_2_ adsorbents toward Dy^3+^, Tb^3+^, and Hg^2+^ ions in aqueous solutions, batch adsorption experiments at pH 4.5 and with a contact time of 2 h were performed. The graphical representations of the adsorption efficiency and adsorption capacity of the prepared samples are depicted in Figure 8. The numerical results are presented in Table 1.

The adsorption results of the Dy^3+^, Tb^3+^, and Hg^2+^ ions are presented only for the functionalized NPs, both γFe_2_O_3_@SiO_2_–NH_2_ and CoFe_2_O_4_@SiO_2_–NH_2_, as adsorption by functionalized NPs is usually much more efficient than adsorption by non-functionalized NPs [46].

The adsorption results showed that both the γFe_2_O_3_@SiO_2_–NH_2_ and the CoFe_2_O_4_@SiO_2_–NH_2_ samples had high adsorption affinity towards Dy^3+^ and Tb^3+^ ions, with relatively high adsorption efficiencies of 83.1% and 89.3%, respectively, but low adsorption capacities of 4.0 mg/g and 4.7 mg/g, respectively.

The adsorption affinity of the γFe_2_O_3_@SiO_2_–NH_2_ and CoFe_2_O_4_@SiO_2_–NH_2_ toward the Dy^3+^, Tb^3+^, and Hg^2+^ ions in the aqueous medium can be explained by Pearson’s hard-and-soft acid-base (HSAB) theory [91]. This concept is based on Lewis’ definition of acids as electron acceptors and bases as electron donors, and it states that soft acids prefer to coordinate and form stronger bonds and more stable complexes with soft bases, whereas hard acids prefer to coordinate and form stronger bonds and more stable complexes with hard bases.

According to the HSAB concept, Dy^3+^ and Tb^3+^ ions are classified as hard Lewis acids, and functional amino (–NH_2_) groups are classified as hard Lewis bases, so Dy^3+^ and Tb^3+^ ions have a high affinity for NH_2_ groups. On the other hand, as a soft Lewis acid, Hg^2+^ is a relatively large (1.02 Å) and polarizable atom, which, in practice, prefers to associate with soft bases. Since Hg^2+^ is larger than Tb^3+^ (0.923 Å) and Dy^3+^ (0.912 Å) and, thus, more polarised, it has a weaker preference for interactions with hard NH_2_ groups. This resulted in its significantly lower adsorption capacity of 2.1 mg/g for the γFe_2_O_3_@SiO_2_–NH_2_ and 1.2 mg/g for the CoFe_2_O_4_@SiO_2_–NH_2_ NPs.

The lower adsorption capacity of Hg^2+^ compared to Tb^3+^ and Dy^3+^, in the context of the HSAB concept, can be explained by the use of the absolute-hardness parameter (*η_s_*). Parr et al. [92] defined the absolute-hardness parameter as *η*_s_ = (*I*_p_ − *A*_s_)/2, where *I*_p_ (eV) is the ionization potential and *A*_s_ (eV) is the electron affinity. Pearson, in a 1988 paper [93], conveniently provided cumulative experimental values for ionization potential and electron affinity, referring to earlier work by Moore [94]. From these values, it is possible to calculate the absolute-hardness parameter (*η*_s_) values, which for Hg^2+^, Tb^3+^, and Dy^3+^ are 5.4 eV, 3.1 eV, and 3.1 eV, respectively. Pearson [93] also defined softness (*σ_s_*) as the inverse of hardness, *σ_s_* = 1/*η*_s_, with zero as the maximum softness. The values of the softness parameter for Hg^2+^, Tb^3+^, and Dy^3+^ are thus 0.19, 0.32, and 0.32, respectively, indicating the softer nature of Hg^2+^ compared to Tb^3+^ and Dy^3+^. Therefore, in this case, a higher complexation affinity of the hard NH_2_ groups for the hard Tb^3+^ and Dy^3+^ ions and a lower complexation affinity for the softer Hg^2+^ is expected, which also agreed with the results of our work.

A possible explanation for the low adsorption capacity of Hg^2+^ is its unusual chemical properties and its character as a soft Lewis acid. The soft nature of Hg^2+^ is related to its ground-state electronic configuration ([Xe]4f^14^5d^10^6s^2^) with filled electron subshells up to 6s, which, due to its stability, strongly resists electron removal, resulting in the very high ionization potential (*I*_p_ 10.434 eV) and moderately high electronegativity (*χ*_P_ 2.00 by Pauling) [95,96] of Hg, which is reflected in its low chemical reactivity [97].

Because all the main energy levels of the Hg atom are filled, and because of the unusually stable 6s^2^ electron pair, Hg can form only very weak hard–soft chemical bonds with amino groups, with a covalent character [96]. Therefore, the interactions of soft Hg with the hard electron-donating N-atom in the NH_2_ group, which is a small (0.16 Å), low-polarizable atom with high electronegativity (*χ*_P_ 3.04 Pauling) and high ionization potential (*I*_p_ 14.534 eV), are not favored [95,98].

In contrast, Tb^3+^ and Dy^3+^, which are hard Lewis acids, according to the HSAB concept, tend to have chemical interactions with the electron-donor N-atoms in the NH_2_ groups, which have the character of hard Lewis bases. This is due to differences in the stability of their electron configurations and electron-density distributions. Compared to Hg, which has a stable electron configuration, Tb ([Xe]6s^2^4f^9^) and Dy ([Xe]6s^2^4f^10^) have stabilized 4f electrons that do not contribute to the formation of chemical bonds, and their chemical behavior is dictated by the 6 =s valence electrons [97]. Despite the presence of 6s^2^ electrons in addition to the 4f and [Xe] nuclei in Tb and Dy, their most stable oxidation state in aqueous media is +3, which makes them more reactive than divalent Hg. Their chemical reactivity gradually decreases from terbium towards mercury in the sequence of Tb > Dy > Hg [97], which is also reflected in their higher affinity for the formation of complexes with NH_2_ groups of APTMS compared to Hg. This is consistent with our finding, in this study, that the adsorption capacities of Tb^3+^, Dy^3+^, and Hg^2+^ decrease in the order of Tb^3+^ > Dy^3+^ > Hg^2+^.

On the other hand, the unexpectedly low adsorption capacity of Tb^3+^ and Dy^3+^ ions, which have the character of hard Lewis acids, is probably also due to the strong hydration of these cations in aqueous solutions and the formation of aqua complexes ([Ln(OH_2_)_9_]^3+^, Ln = Tb, Dy), which is also reflected in their high hydration-enthalpy values for Tb^3+^ (Δ*H*_hydr_ −3540 kcal/mol) and Dy^3+^ (Δ*H*_hydr_ −3570 kcal/mol) [99,100,101]. Such aqua complexes are easily formed because of excess water, and they are prone to substitution reactions, in which water molecules are successively replaced by amino ligands and vice versa [102].

Bjerrum [103] determined that a metal complex in an aqueous solution is formed by the exchange of a coordinated water molecule directly bound to the central lanthanide ion (Ln^3+^) with other ligands, provided that the ligand has a sufficiently strong affinity for the lanthanide ion to compete with the affinity of the coordinated water. Such exchanges result in the formation of strong complexes with inner hydration shells [104]. When the ligand replaces the water molecule of the aqua-complex ion, a new metal complex is formed and equilibrium is established. It is assumed that this formation does not take place in one step, but in several steps, involving the following: (i) the joint diffusion of the hydrated cation and anion, (ii) the partial loss of solvent to form the ion pair, (iii) the loss of water from the first coordination sphere of the cation, and (iv) the formation of the complex species. The rate-determining step is the loss of the water molecule from the coordination sphere of the Ln^3+^ ion and, thus, depends only on the hydration properties of the Ln^3+^ ion [105,106].

In general, a maximum number of water molecules are distributed around Ln^3+^ ions during the hydration process, depending on the size of the Ln^3+^ ion and its electronic properties. It is known that the ionic radii of Ln^3+^ ions in the Ln species decrease as the atomic number increases due to lanthanide contraction, which is a consequence of the incomplete mutual protection of the valence f-orbitals.

According to the ratio of the radii of the Ln^3+^ ions to the radii of the oxygen atoms (1.34 Å) in coordinated water molecules (*r*_ion_/*r*_0_), all the hydrated Ln^3+^-ions in aqueous solutions occupy the configuration of a tricapped trigonal prismatic [107] geometry with six nearly identical water molecules at the vertices of the trigonal prism and the remaining three water molecules capping the prism faces [108].

Hydrates of the lighter Ln^3+^ ions (La^3+^-Nd^3+^) have a regular tricapped trigonal prismatic configuration, with slightly longer bond distances from the Ln^3+^ ion to the capping water molecules (Ln-O) than to the water molecules forming the prism. The decrease in the radii of the Ln^3+^-ions with the increase in the atomic number of the Ln-species starting from Nd^3+^ does not, in principle, affect the structure of the prism, but has a strong effect on the more weakly bound capping positions of the water molecules in the prismatic structure, resulting in a partial loss of the water molecules in the capping positions for the heaviest Ln^3+^-ions (Ho^3+^-Lu^3+^). In fact, studies have shown that the bond strength of the three-capping water molecules is strong at the beginning of the Ln series for nonahydrates (e.g., La^3+^-Sm^3+^), while in the Ln series starting from (Sm^3+^-Lu^3+^), the Ln–O capping bonds become weaker and shorter at the same time. The Ln–O change in the [Ln(OH_2_)_9_]^3+^ for Ln^3+^-ions (e.g., La^3+^, Sm^3+^, Tb^3+^, Dy^3+^, Ho^3+^, and Lu^3+^) occurs in the following sequence: La-O (2.52 Å) > Sm-O (2.46 Å) > Tb-O (2.39 Å) > Dy–O (2.37 Å) > Ho–O (2.36 Å) > Lu–O (2.31 Å) [109]. Consequently, in the lanthanide series starting from Sm^3+^ onward, the three water molecules in the structure are not equally strongly bound and one of the water molecules is at a shorter distance from the Ln-center than the other two. In the series from Ho^3+^ to Lu^3+^, the water deficit and the differences between the strongly bound water molecules and the more weakly-bound molecules are even greater [108,109]. 

Therefore, since Ln-O bonds are more easily broken, in the whole Ln series, the lighter lanthanide Ln^3+^ -ions (La^3+^–Nd^3+^) form stable nonahydrates, while the heavier Ln^3+^-ions (Ho^3+^–Lu^3+^) form octahydrates. Intermediate Ln^3+^-ions (Sm^3+^–Dy^3+^) favor the formation of complex forms of nona- and octahydrates with non-integer coordination numbers (CN) between 8 and 9. Therefore, the CN of these intermediate lanthanides should be average with respect to the ratio of nona- to octahydrate forms [110,111]. 

Such Ln-O bond behavior determines the hydration behavior of Ln^3+^ ions and explains their very unusual and complex ligand exchange kinetics throughout the Ln series [110,111]. The peculiarity is that the exchange rate of water molecules between the first hydration shell and the bulk solvent increases in the direction from La^3+^ to Gd^3+^, reaches its maximum in the central region of the Ln series (Tb^3+^, Dy^3+^), and then decreases up to Lu^3+^ [112,113]. The physical reasons for these phenomena are still not well understood and are the subject of many investigations [110,111].

On the other hand, H_2_O is also known to be a hard base [114], which, in accordance with the HSAB concept and the above values of absolute hardness, associates with hard ligands rather than soft ligands, because, in this case, hard–hard interactions are more favorable. Since the amino (–NH_2_) group is a hard Lewis base, the substitution of the amino ligand and H_2_O is relatively favorable from this point of view [100,114], which makes lanthanide-ion complexes of Ln^3+^ (Ln = Tb, Dy) extremely labile [115]. In contrast, for transition metals, the lability of complexes generally varies with their electronic configuration. Some complexes are labile, while others are kinetically very inert, such as d^3^ species and low-spin d^6^ species, with high stabilities and high activation energies for ligand substitution [115].

The formation of amino complexes is largely related to the availability of active sites on the adsorbent surfaces and the coordination number. The two key factors influencing the coordination number and complex formation, as well as their chemical stability, areas follows: (i) the influence of the donor N-atoms in the immediate vicinity of the metal ion (Tb^3+^, Dy^3+^, Hg^2+^), which, due to interatomic tension, prevent more N-atoms from making contact with the metal ion; and (ii) the steric repulsions between the larger substituent groups in the APTMS, which are bonded to the donor N-atom (i.e., H_2_N(CH_2_)_3_-), which determine how much of the ligand can be surrounded by the metal ion [115]. The lack of available active sites thus reduces the ability to form complexes, resulting in a lower adsorption capacity. 

The electron-donating N-atoms of the amino groups of APTMS in the surface coating of the adsorbent possess a free-electron pair that can be donated to form a coordination bond with the Tb^3+^, Dy^3+^, and Hg^2+^ metal ions in the aqueous medium. Depending on the coordination number, which is 9 for Tb^3+^ and Dy^3+^ [98,116] and 6 for Hg^2+^ [117,118], these metal ions can coordinate linearly with one or two amino groups, with the remaining coordination sites occupied by water molecules. Thus, the coordination mechanism of Tb^3+^, Dy^3+^, and Hg^2+^ with the amino groups of APTMS on the surfaces of γFe_2_O_3_@SiO_2_–NH_2_ and CoFe_2_O_4_@SiO_2_–NH_2_ adsorbents can be represented as [101,119,120]:

RNH_2_ + Ln^3+^ ⇔ [Ln(RNH_2_)(H_2_O)_8_]^3+^ (Ln = Tb, Dy) (1)

RNH_2_ + Hg^2+^ ⇔ [Hg(RNH_2_)(H_2_O)_5_]^2+^ (2)

After the adsorption of Tb^3+^, Dy^3+^, and Hg^2+^, the possibility of recovering both adsorbents, γFe_2_O_3_@SiO_2_–NH_2_ and CoFe_2_O_4_@SiO_2_–NH_2_, for the reuse and recycling of Tb^3+^, Dy^3+^, and Hg^2+^ ions were verified by a desorption process. The choice of agent for desorption is based on the type of adsorbate–adsorbent system [121] and depends mainly on the compatibility between adsorbate and adsorbent, the pH and ionic strength of the medium, the complexation ability, the desorption-agent content, and the exposure time, as these variables can modify the desorption behavior or destroy the adsorbent structure [121]. In addition, desorption phenomena are related to a series of surface interactions and diffusion into micropores or the intraparticle spaces of the adsorbents [99].

**Figure 9:**
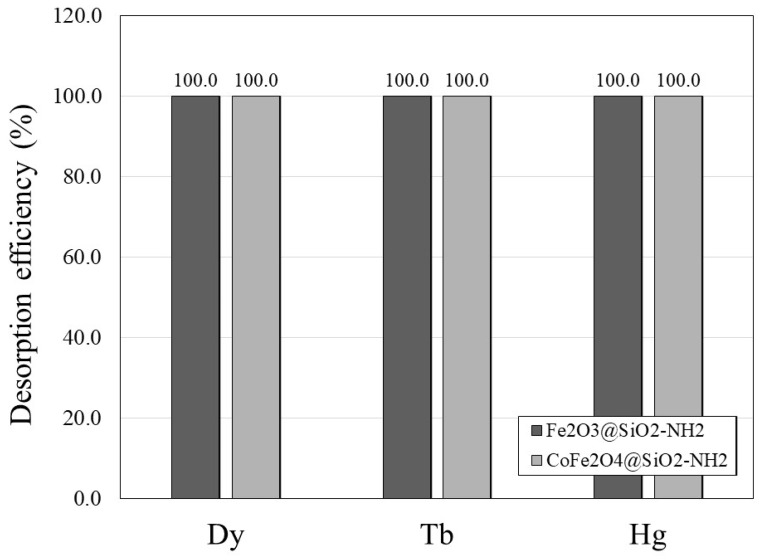
Desorption efficiency of γFe_2_O_3_@SiO_2_–NH_2_ and CoFe_2_O_4_@SiO_2_–NH_2_ NPs.

In our previous research [122,123], we studied some desorption conditions and the use of different acidic desorption agents, such as hydrochloric acid (HCl), nitric acid (HNO_3_), and citric acid, and we found that HNO_3_ gave better results than the other two desorption agents. Therefore, in the present study, a desorption procedure for Tb^3+^, Dy^3+^, and Hg^2+^ was carried out using a 1-M aqueous solution of HNO_3_ for 1 h at room temperature and with pH 4.5 [124]. The desorption was carried out in one cycle only due to the loss of material during the desorption process. The results of the desorption efficiency are shown graphically in Figure 9. After 1 h, the Tb^3+^, Dy^3+^, and Hg^2+^ ions were completely desorbed from the surfaces of the γFe_2_O_3_@SiO_2_–NH_2_ and CoFe_2_O_4_@SiO_2_–NH_2_ adsorbents, indicating the potential for the stable reusability of the prepared adsorbents and the high potential of these adsorbents for the recycling and removal of heavy metals.

Table 2 shows the comparison of the adsorption of the Dy^3+^, Tb^3+^, and Hg^2+^ ions onto the prepared γFe_2_O_3_@SiO_2_–NH_2_ and CoFe_2_O_4_@SiO_2_–NH_2_ NPs with the adsorption of Dy^3+^, Tb^3+^, and Hg^2+^ on different adsorbents. It can be observed that the adsorption conditions, such as the initial adsorbate concentration (*c*_ads,0_), adsorbent dosage (*γ*_ads_), time of adsorption (*t*_ads_), adsorption temperature (*T*_ads_), and solution pH, were very different, making the comparison of the adsorption performances a difficult and a complex task.

Su et al. [125] studied the adsorption capacity of Dy^3+^ with the adsorbate Fe_3_O_4_@SiO_2_@polyaniline-graphene oxide, and they demonstrated an adsorption capacity of 16.0 mg/g, an adsorption efficiency of 98%, and a desorption efficiency of 95% at pH 4. Liu et al. [126] reported an adsorption capacity of 28.3 mg/g, an adsorption efficiency of >80%, and a desorption efficiency of >95% for Dy^3+^ at pH 7 using Fe_3_O_4_-C_18_–chitosan–DETA. With the same pH of 7, using NH_4_OH@SiO_2_ (APTMS), Kegl et al. [84] reported an adsorption capacity of 23.2 mg/g and an adsorption efficiency of 94% for Dy^3+^. Javadian et al. [127] reported the adsorption characteristics of synthetic-polymer-based magnetic adsorbent (M-PPTA) toward Dy^3+^ at pH 5.5, and found an adsorption capacity of 24.0 mg/g, an adsorption efficiency of 98.4%, and a desorption efficiency of >84%. Shinozaki et al. [128] noted 18.4 mg/g of adsorption capacity and 30% of adsorption efficiency for Dy^3+^ at pH 1 using an adsorbate of polymeric adsorbents modified with ethylenediamine (EDA) and diglycolamic acid (DGA).

Alcaraz et al. [129] reported the use of chemically and physically activated carbons from spent-coffee waste to adsorbed Dy^3+^ ions. They showed an adsorption capacity of chemically activated carbons toward Dy^3+^ of 31.26 mg/g and an adsorption efficiency of Dy^3+^ of 96% at pH 4. When using physically activated carbons from spent-coffee waste, an adsorption capacity of 33.52 mg/g and an adsorption efficiency of 99% were obtained. Further, Viana et al. [130] reported an adsorption capacity of 0.570 mg/g and an adsorption efficiency of 89% for Dy^3+^ using *ulva lactuca*—Chlorophyta (green), while an adsorption capacity of 0.526 mg/g and an adsorption efficiency of 84% were obtained for Dy^3+^ by using *Gracilaria* sp.—Rhodophyta (red). According to Zhang et al. [131], an adsorption capacity of 1.85 mg/g and an adsorption efficiency of 84% of Dy^3+^ at pH 3 were obtained using an Fe^0^–SiO_2_–PA/SiO_2_–DTPA adsorbent.

Su et al. [125], Zhang et al. [131], Barros et al. [132], Tong et al. [133], and Kegl et al. [134] studied the adsorption capacity of Tb^3+^ using various adsorbent materials under different adsorption conditions. Using graphene oxide at pH 4, Su et al. [125] obtained an adsorption capacity of 11.8 mg/g, an adsorption efficiency of 98%, and a desorption efficiency of 95% for Tb^3+^. Zhang et al. [131] used an Fe^0^–SiO_2_–PA/SiO_2_–DTPA adsorbate at a pH of 3 and obtained an adsorption capacity of 1.4 mg/g for Tb^3+^. Barros et al. [132] reported the use of molecular-sieve zeolite for the adsorption of Tb^3+^ ions at a pH of 5, and they noted an adsorption capacity of 2.59 mg/g alongside an adsorption efficiency of 80%, and a desorption efficiency of >60% for Tb^3+^; furthermore, they obtained an adsorption capacity of 5.07 mg/g for the Tb^3+^ using *B. cereus* biomass-supported zeolite adsorbate. Tong et al. [133] used multi-walled carbon nanotubes with tannic acid (TA-MWCNTs) for the adsorption of Tb^3+^ at pH 5 and obtained an adsorption capacity of 8.55 mg/g, while Kegl et al. [134] used a superparamagnetic γ-Fe_2_O_3_-NH_4_OH@SiO_2_ (APTMS) adsorbent and obtained an adsorption capacity of 0.204 mg/g for the adsorption of Tb^3+^ ions from water at a pH of 7.

Adsorption studies on Hg^2+^ ions with CoFe_2_O_4_–chitosan–graphene, polypyrrole-functionalized magnetic kaolin (Ppy-Fe_3_O_4_/kaolin), and CoFe_2_O_4_@SiO_2_–NH_2_ adsorbents at a pH of 7 showed adsorption capacities of 361.0 mg/g, 255.2 mg/g, and 149.3 mg/g, respectively. These studies were performed by Zhang et al. [135], Lin et al. [136], and Wang et al. [137], respectively.

In recent works, Xia et al. [138], Allwin Mabes Raj et al. [122], and Inglezakis et al. [139] studied the adsorption of Hg^2+^ using CoFe_2_O_4_@SiO_2_–EDTA, γ–Fe_2_O_3_@NH_2_, and Fe_3_O_4_ as adsorbates, and they noted adsorption capacities of 103.3 mg/g, 85.6 mg/g, and 28.0 mg/g for Hg^2+^, respectively. Liu et al. [140] studied the adsorption capacity of Hg^2+^ with the adsorbate rice-husk-activated carbon (RHAC) and demonstrated an adsorption capacity of 55.87 mg/g at a pH of 5. Denizli et al. [141] studied the adsorption of Hg^2+^ with magnetic poly (vinyl alcohol)—procion blue MX-3G as the adsorbent and noted an adsorption capacity of 69.2 mg/g, an adsorption efficiency of >94%, and a desorption capacity of 95% for Hg^2+^. An adsorption study of Hg^2+^ with amino-functionalized SiO_2_ particles (NH_2_@SiO_2_) conducted by Raj et al. [123] and a study with activated carbon performed by Solis et al. [142] showed adsorption capacities of 3.75 mg/g and 2.5 mg/g, respectively.

Nevertheless, it can be observed that the adsorption of Dy^3+^, Tb^3+^, and Hg^2+^ ions is tested mostly in acidic or neutral aqueous media, and that the adsorption capacity is higher in surface-functionalized adsorbent nanomaterials. In addition, some adsorbents have significantly higher adsorption properties for Hg^2+^ than for Dy^3+^ and Tb^3+^ ions compared to our prepared adsorbent materials. However, the main advantages of our adsorbents are their relatively fast kinetics of adsorption, which are associated with their nano-size and functionality, their high desorption efficiency, and the convenient and highly efficient sustainable recovery of the used adsorbate material at the end of the adsorption process by magnetic attraction.

### 2.3. Cytotoxicity Study

**Figure 10:**
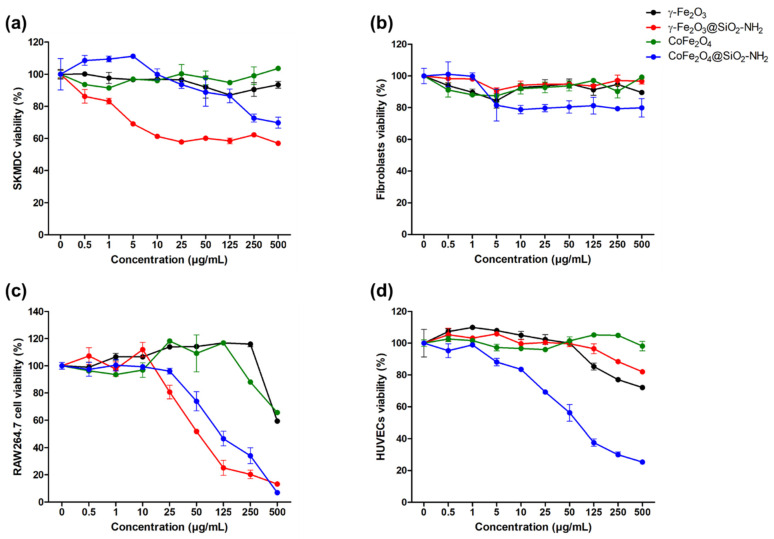
Cell viability (%) of (**a**) SKMDCs, (**b**) fibroblasts, (**c**) RAW264.7, and (**d**) HUVECs treated with different concentrations of nanoparticles for 3 days. Results are presented as mean ± SEM, n = 3.

The cytotoxicity of the nanoparticles was tested in different healthy cell lines after 3 days of incubation. In the SKMDCs, the γFe_2_O_3_ nanoparticles showed lower toxicity than the γFe_2_O_3_@SiO_2_–NH_2_, as shown in Figure 10a. The cell viability reached 69 ± 0.72% after 3 days of incubation with 5 µg/mL of γFe_2_O_3_@SiO_2_–NH_2_, which decreased to 57 ± 0.47% at a concentration of 500 µg/mL. Similarly, the CoFe_2_O_4_ nanoparticles showed lower toxicity than the CoFe_2_O_4_@SiO_2_–NH_2_. The incubation of the SKMDCs with CoFe_2_O_4_@SiO_2_–NH_2_ up to a concentration of 125 µg/mL for 3 days showed lower toxicity with a cell viability above 80%; however, the cell viability decreased to 70 ± 3.41% at 500 µg/mL.

In the fibroblasts, all the nanoparticles showed lower toxicity when the cell viability was above 80%, with concentrations of up to 500 µg/mL (Figure 10b).

In the macrophage RAW264.7 cell line, both the γFe_2_O_3_ and the CoFe_2_O_4_ showed lower toxicity than the γFe_2_O_3_@SiO_2_–NH_2_ and CoFe_2_O_4_@SiO_2_–NH_2_ (Figure 10c). At 50 µg/mL, the cell viability reached 52 ± 0.61% and 74 ± 7% for the γFe_2_O_3_@SiO_2_–NH_2_ and CoFe_2_O_4_@SiO_2_–NH_2_, respectively. However, at 500 µg/mL, the cell-viability values were 59 ± 0.10%, 13 ± 0.51%, 66 ± 0.31%, and 7 ± 0.23% for the γFe_2_O_3_, γFe_2_O_3_@SiO_2_–NH_2_, CoFe_2_O_4_, and CoFe_2_O_4_@SiO_2_–NH_2_, respectively. It is worth mentioning that the macrophage RAW264.7 cells were more sensitive to the γFe_2_O_3_@SiO_2_–NH_2_ and CoFe_2_O_4_@SiO_2_–NH_2_ than the other cell lines.

The toxicity of the nanoparticles was also tested in the HUVECs (Figure 10d). The results showed the low toxicity of the γFe_2_O_3_, γFe_2_O_3_@SiO_2_–NH_2_, and CoFe_2_O_4_ compared with the CoFe_2_O_4_@SiO_2_–NH_2_, which showed a decrease in cell viability (69 ± 1.26%) at 25 µg/mL, reaching 25 ± 0.86% at 500 µg/mL.

**Figure 11:**
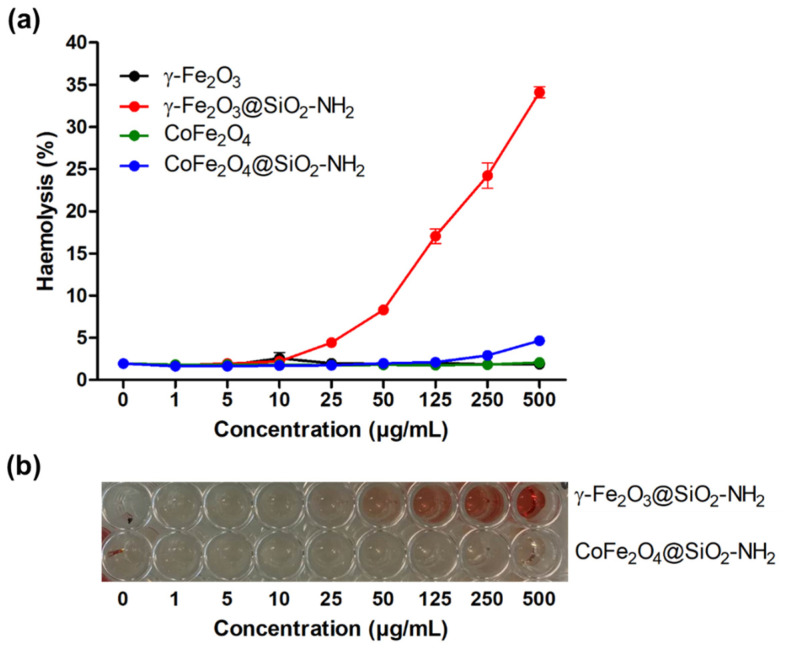
In vitro hemolytic studies. (**a**) Hemolytic effect of different nanoparticles on human blood at different concentrations (**a**), representative image of the hemolytic effect (red-colored supernatant) of nanoparticles (**b**).

The results presented in Figure 11a show that none of the nanoparticles had hemolytic effects, except the nanoparticles of γFe_2_O_3_@SiO_2_–NH_2_, which showed a dose-dependent hemolytic effect. A change in the supernatant color was observed by the naked eye at concentrations of 50 µg/mL and above, indicating the lysis of red blood cells and hemoglobin release (Figure 11b) [5].

**Figure 12:**
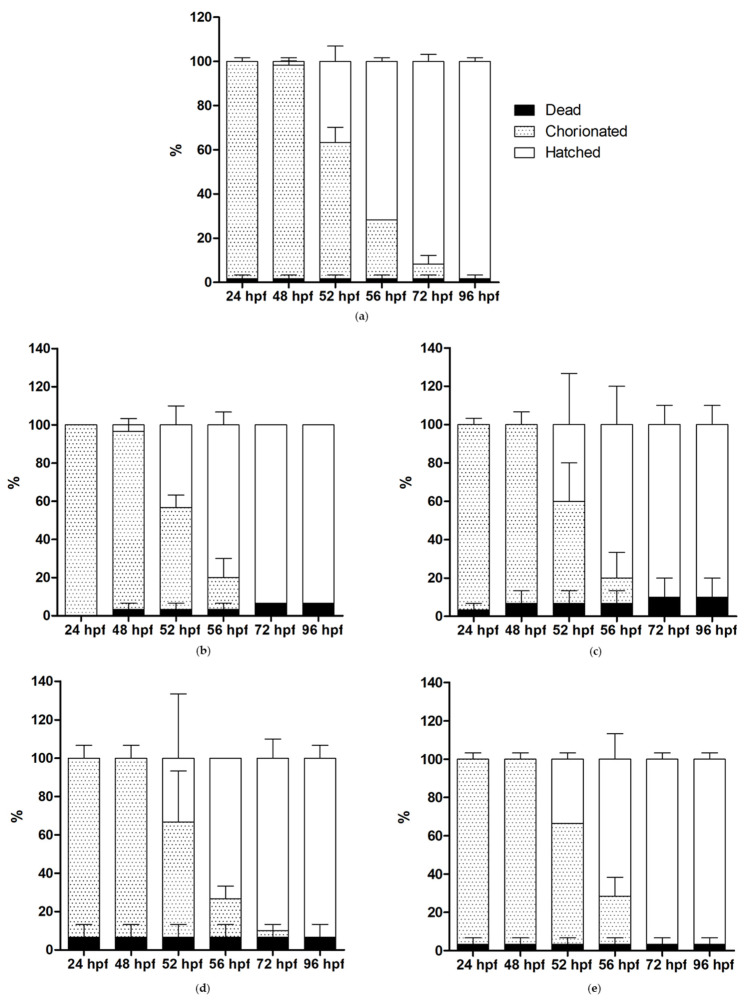
Zebrafish-embryo development expressed as percentages of dead, chorionated, and hatched, in water containing concentration of 500 mg/L of nanoparticles. Control group (**a**) is of growth without the use of any NPs (**b**) γFe_2_O_3_ (**c**) γFe_2_O_3_@SiO_2_–NH_2_ (**d**) CoFe_2_O_4_, and (**e**) CoFe_2_O_4_@SiO_2_–NH_2_ for 24, 48, 52, 56, 72, and 96 h post-fertilization (hpf). Data are presented as mean ± SEM of two independent experiments.

A toxicity study on zebrafish embryos exposed to different concentrations of nanoparticles as shown in Figure S3 revealed that none of the nanoparticles had toxic effects on the zebrafish embryos until 96 hpf compared to the control, even at a high concentration of 500 mg/L (Figure 12).

Table S1 depicts the list of various adsorbent materials with their toxicological assessment in different biological systems. Many studies investigating the toxicities of different materials using in vivo and in vitro studies, such as a toxicity study of human kidneys (HEK293) using magnetic, SiO_2_-coated nanoparticles with an exposure of up to 1.0 μg/μL for 12 h, which was reported by Shim et al. [143]. In their SiO_2_ in vitro study, Pisani et al. [144] used A549 (human) lung cells with 0.1–6-μg/cm^2^ dosages and exposed them for 24 h. A toxicity study was reported by Ellinger-Ziegelbauer and Pauluhn [145] on rat-lung cells with MWCNT at a 11-mg/m^3^ concentration for a 6-h (aerosol) 90-day post-exposure period. Jovanović et al. [146] studied the TiO_2_ (anatase) and hydroxylated fullerene toxicity of 40-μg/mL fullerenes and 170 ng/mL in Danio rerio (embryo). With 2.5 μg/mL and 25 μg/mL of Ag in human colon cells, toxicity was studied by Böhmert et al. [147]. Conde et al. [148] studied toxicity using Au, functionalized with anti-sense cDNAs at dosages of 30 nM in the HCT-116 (human) colon.

Our present study investigating the cytotoxicity of iron-oxide NPs generally showed that both these NPs, γFe_2_O_3_@SiO_2_–NH_2_ and CoFe_2_O_4_@SiO_2_–NH_2_, are non-toxic.

## 3. Materials and Methods

All the chemicals used in this study were generally of reagent grade, obtained from commercial sources without further purification: iron (II) chloride tetrahydrate (FeCl_2_·4H_2_O, 98%, 198.81 g/mol, CAS no. 13478-10-9, Sigma-Aldrich, Merck Group KGaA, Darmstadt, Germany), iron (III) chloride hexahydrate (FeCl_3_·6H_2_O, ≥98%, 270.3 g/mol, CAS no. 10025-77-1, Sigma-Aldrich, Merck Group KGaA, Darmstadt, Germany), cobalt (II) chloride hexahydrate (CoCl_2_·6H_2_O, 98%, 237.93 g/mol, CAS no. 7791-13-1, Sigma-Aldrich, Merck Group KGaA, Darmstadt, Germany), ammonium hydroxide aqueous solution (NH_4_OH, 25%, 35.05 g/mol, 0.91 g/mL, CAS no. 1336-21-6, GramMol, Zagreb, Croatia), sodium hydroxide (NaOH, ≥98% (anhydrous), 40 g/mol, CAS no. 1310-73-2, Sigma-Aldrich, Merck Group KGaA, Darmstadt, Germany), potassium hydroxide (KOH, 1 mol/L (1-N), Titripur^®^, 56.11 g/mol, 1.05 g/mL, CAS no. 1310-58-3, Sigma-Aldrich, Merck Group KgaA, Darmstadt, Germany), potassium chloride (KCl, ACS reagent, 99.0–100.5%, 74.55 g/mol, CAS no. 7447-40-7, Sigma-Aldrich, Merck Group KgaA, Darmstadt, Germany), hydrochloric acid (HCl, for 1000 mL, 1 mol/L (1-N), Titrisol^®^, 36.46 g/mol, 1.09 g/ml, CAS no. 7647-01-0, Sigma-Aldrich, Merck Group KgaA, Darmstadt, Germany), nitric acid (HNO_3_, ACS reagent, 70%, 63.01 g/mol, 1.413 g/mL, CAS no. 7697-37-2, Sigma-Aldrich, Merck Group KgaA, Darmstadt, Germany), 2-propanol (C_3_H_8_O, 99.8%, 60.1 g/mol, 0.785 g/mL, CAS no. 67-63-0, GramMol, Zagreb, Croatia), ethanol (C_2_H_5_OH, 96%, 46.07 g/mol, 0.810 g/mL, CAS no. 64-17-5, GramMol, Zagreb, Croatia), tetraethyl orthosilicate TEOS (C_6_H_20_O_4_Si, 99%, 208.33 g/mol, 0.94 g/mL, CAS no. 78-10-4, Sigma-Aldrich, Merck Group KGaA, Darmstadt, Germany), 3-aminopropyltrimethoxysilane APTMS (C_6_H_17_NO_3_Si, 97%, 179.29 g/mol, 1.027 g/mL, CAS no. 13822-56-5, Sigma-Aldrich, Merck Group KGaA, Darmstadt, Germany), terbium (III) chloride hexahydrate (TbCl_3_∙6H_2_O, 99.9%, 373.38 g/mol, CAS no. 13798-24-8, Sigma-Aldrich, Merck Group KGaA, Darmstadt, Germany), dysprosium (III) nitrate pentahydrate (Dy(NO_3_)_3_∙5H_2_O, 99.9%, 438.59 g/mol, CAS no. 10031-49-9, Sigma-Aldrich, Merck Group KGaA, Darmstadt, Germany), and mercury (II) nitrate monohydrate (Hg(NO_3_)_2_∙H_2_O, ≥98.5%, 342.62 g/mol, CAS no. 7783-34-8, Sigma-Aldrich, Merck Group KGaA, Darmstadt, Germany). For the preparation of all suspensions and solutions, deionized water (dH_2_O) was used.

### 3.1. Synthesis of Magnetic Nanoparticles (MNPs)

Spinel-type MNPs of maghemite (γ-Fe_2_O_3_) and Co-ferrite (CoFe_2_O_4_) were obtained by co-precipitation of M^2+^ (M = Fe, Co) and Fe^3+^ salts at slightly elevated temperature in an alkaline aqueous medium according to Schikorr reaction [149]:

M^2+^ + 2Fe^3+^ + 4OH^−^ + O_2_ = MO∙Fe_2_O_3 (s)_↓ + 2H_2_O (3)

#### 3.1.1. γ-Fe_2_O_3_ NPs

For the synthesis of γFe_2_O_3_ NPs, 50 mL of 25% NH_4_OH in a round-bottomed reaction flask was heated up to (87 ± 2) °C by reflux and stirred at 400 rpm. A 0.5-M aqueous solution of Fe^2+^ and Fe^3+^ in a molar ratio of 1:2 was added to the ammonia solution until pH 10 was reached. The reaction was carried out for 1 h at (87 ± 2) °C. Instantly, when the two solutions were mixed, a dark-brown precipitate of magnetic phase was formed. After the reaction, the dark-brown γFe_2_O_3_ precipitate was separated from the supernatant by settling on the permanent magnet and rinsed with deionized water several times. Finally, the rinsed precipitate was dried in a laboratory oven at 90 °C for 24 h.

#### 3.1.2. CoFe_2_O_4_ NPs

For the synthesis of CoFe_2_O_4_ NPs, stock solutions containing Co^2+^ and Fe^3+^ ions were prepared using CoCl_2_·6H_2_O and FeCl_3_·6H_2_O as source materials. Stoichiometric amounts of the appropriate chlorides were dissolved in deionized water. The concentration of the solution was 0.5 M in all experiments, referred to as chloride concentration. The solution was then hydrolyzed in a 0.5-M aqueous solution of sodium hydroxide preheated to (87 ± 2) °C by reflux and stirred at 400 rpm. The reaction was carried out for 1 h at pH 10. After the reaction was completed, the dark-brown CoFe_2_O_4_ precipitate was separated from the supernatant by settling on the permanent magnet and rinsed with deionized water. The rinsing procedure was repeated several times, and the rinsed precipitate was finally dried in a laboratory oven at 90 °C for 24 h.

#### 3.1.3. γ-Fe_2_O_3_@SiO_2_-NH_2_ and CoFe_2_O_4_@SiO_2_-NH_2_ NPs

For the in situ preparation of γ-Fe_2_O_3_@SiO_2_–NH_2_ and CoFe_2_O_4_@SiO_2_–NH_2_ core@shell NPs, 21.6 mol% of 2-propanol, 15.1 mol% of distilled water, 2.2 mol% of 25% NH_4_OH solution, 4 mL of the prepared γ-Fe_2_O_3_ or CoFe_2_O_4_ colloidal suspension with a mass concentration (*γ_i_*) 1.035 ± 0.005 g/mL, 0.25 mol% of TEOS, and 0.36 mol% of APTMS were mixed under magnetic stirring at 500 rpm in a closed vessel for 24 h at room temperature. After the reaction finished, the sediment was rinsed several times with ethanol (96 wt.%) and distilled water. The obtained core@shell superparamagnetic NPs were separated from the supernatant by using the permanent external magnet and dried overnight in the laboratory oven at 90 °C.

**Figure 13:**
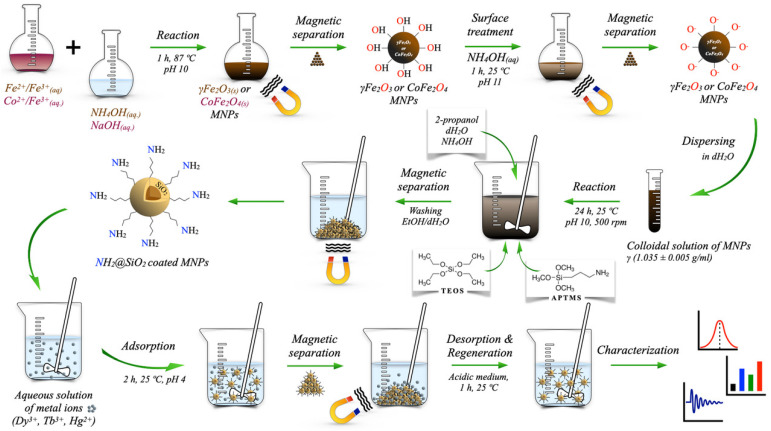
Schematic representation of the experimental procedure.

The experimental procedure for this study is schematically presented in Figure 13.

### 3.2. Characterization of MNPs

Prepared samples were characterized using X-ray diffractometry and transmission-electron microscopy in combination with energy-dispersive X-ray spectroscopy, Brunauer–Emmet–Teller specific-surface-area technique, Fourier-transform infrared spectroscopy, thermogravimetric analysis, electro-kinetic (ξ) potential measurements, inductively coupled plasma atomic emission spectroscopy, and vibrating-sample magnetometry.

#### 3.2.1. X-ray Diffractometry (XRD)

We used X-ray diffractometry (XRD) for structural analysis with a Brucker D4 Endeavor X-ray diffractometer coupled with CuK_α_ radiation (Bruker D4 Endeavor, Bruker, Billerica, MA, USA). The measurements were performed at room temperature with a time step of 30 s within the range of Bragg’s angle 2 *θ* from 20° to 80°, with an angle step of 0.036°. The XRD utilized a Cu anode with a wavelength of 0.154 nm.

#### 3.2.2. Transmission-Electron Microscopy (TEM) with Energy-Dispersive X-ray Spectroscopy (EDXS)

The TEM images were taken using a JEOL JEM-2100 microscope, operated by drop-casting the nanoparticle suspensions on the thin carbon-coated copper grid (200 mesh, holly carbon) and drying under ambient conditions. The EDXS analyses were performed at 200 kV using a JEOL JEM-2010 microscope (JEM 2100 JEOL, JEOL Ltd., Musashino Akishima, Tokyo, Japan).

#### 3.2.3. Fourier-Transform Infrared Spectroscopy (FT-IR)

The FT-IR data were collected using a Spectrum Two FT-IR Spectrometer (PerkinElmer, Waltham, MA, USA) utilizing a KBr window for data collection over a spectral range of 400 cm^−1^ to 4000 cm^−1^ at a resolution of 0.5 cm^−1^. The FT-IR spectra were recorded with PerkinElmer’s Spectrum 10™ software at room temperature in the transmittance mode.

#### 3.2.4. Brunauer–Emmet–Teller Method (BET)

The BET was used to determine the specific surface areas of NPs by using Micromeritics, Flow Prep 060, with Tristar II 3020 (Micromeritics Instrument Corporation, Norcross, GA, USA). All samples were degassed at 110 °C for 24 h prior to each measurement. The specific surface area was measured in the 0.05–0.3 range of relative pressure in nitrogen gas at a temperature of 77.35 K after 24 h.

#### 3.2.5. Thermogravimetric Analysis (TGA)

To predict the thermal stability and chemical degradation of the functional groups grafted to the surfaces of the NPs, a TGA analysis was performed using a PerkinElmer TGA4000 thermogravimetric analyzer (PerkinElmer, Waltham, MA, USA) calibrated with nickel and iron as Curie-point-reference materials.

For the experiments, prepared powdered sample specimens were placed in a corundum ceramic sample pan, and the weights of these specimens ranged between 2 mg and 50 mg. The experiments were conducted by continuously monitoring the mass of a sample in nitrogen purge gas at a flow rate of 20 mL/min and a heating rate of 10 °C/min over a temperature range of 30 °C to 900 °C, and were controlled by PerkinElmer’s thermal software Pyris Software™ version 10.1.

#### 3.2.6. Electro-Kinetic (ξ)-Potential Measurements

Dynamic light scattering was used to determine electro-kinetic phenomena, which involve the interrelation between mechanical and electrical effects at a moving interface. Electro-kinetic results were expressed in terms of ζ-potential, determined from electrophoretic mobility of particles through a field with known strength, and the term of isoelectric point (IEP), referring to the conditions under which the ζ-potential is zero. When pH is equal to or close to the isoelectric point, NPs tend to be unstable, form clusters, and precipitate. The ζ-potential was measured by ZetaSizer Nanoseries Malvern Instruments (Malvern Panalytical Ltd., Spectris Group, London, UK). Aqueous solutions of NaOH and HCl were employed to adjust the pH values of suspensions. All measurements were performed at room temperature.

#### 3.2.7. Potentiometric Titration

The pH potentiometric titrations were used for the determination of the total charge of aqueous colloidal dispersions of MNPs. The titrations were carried out in forward (acidic-to-alkaline) and backward (alkaline-to-acidic) directions at 2.5 < pH < 11.0 using 0.1-M-HCl and 0.1-M-KOH aqueous solutions as titrants. A two-burette instrument, Mettler T-70 (Mettler Toledo, Columbus, OH, USA), was used. It was equipped with a combined glass-electrode Mettler T DG 117. The burettes were filled with 0.1 M HCl and 0.1 M KOH. All the solutions were prepared with distilled H_2_O with a carbonate content <10^−6^ mol/L, which was achieved through boiling and consequent cooling in a nitrogen atmosphere.

Prior to the titration, the ionic strength was adjusted to an approximate value of 0.1 mol/L by the addition of a 3-M-KCl aqueous solution and then maintained constantly within 2% of the initial value upon the addition of HCl and KOH solutions.

The samples were titrated in forward and backward runs between pH 2 and pH 11. After each addition, the volume of the titration reagent was read when the equilibrium condition <0.1 mV/min was reached or the condition of the maximum waiting time of 3 min was satisfied. The blank HCl–KOH titrations were performed under the same conditions as mentioned above [150].

The titrant volume was normalized to the mass of the titrated samples and expressed as a charge per mass Q/m (in mmol/g) vs. pH curve. The amounts of charged NH_2_ surface groups in the products were expressed in mmol/g sample. The determination of the amount of charged functional groups is described in detail elsewhere [90,150,151].

#### 3.2.8. Vibrating-Sample Magnetometry (VSM)

For magnetization measurements, a Lake Shore 7400 vibrating-sample magnetometer was used (Lake Shore Cryotronics, Inc, Westerville, OH, USA). The mass magnetization *M* (emu/g) as a function of the applied magnetic field *H* (Oe) was measured at room temperature for all the prepared samples.

### 3.3. Adsorption and Desorption Tests for Dy^3+^, Tb^3+^ and Hg^2+^ Ions

To evaluate the affinity of Dy^3+^, Tb^3+^, and Hg^2+^ ions to the surfaces of the prepared γFe_2_O_3_@SiO_2_–NH_2_ and CoFe_2_O_4_@SiO_2_–NH_2_ adsorbent NPs, 20 mL of 10-mM standard aqueous solutions was prepared from TbCl_3_∙6H_2_O, Dy(NO_3_)_3_∙5H_2_O, and Hg(NO_3_)_2_∙H_2_O at pH 4 and temperature of 25 °C.

The adsorption study was performed by separate mixing of 50 mg γFe_2_O_3_@SiO_2_-NH_2_ and CoFe_2_O_4_@SiO_2_–NH_2_ adsorbents with the prepared aqueous solutions of Dy^3+^, Tb^3+^, and Hg^2+^ ions with a concentration of 10 mM at a temperature of 25 ºC, for an adsorption time of 2 h. After the adsorption of Dy^3+^, Tb^3+^, and Hg^2+^ ions, the magnetic adsorbents were removed from aqueous solutions with an external permanent magnet. To determine the adsorption efficiency and capacity, the ICP-OES method was used (ICP-OES, SPECTRO CITROS VISION, SPECTRO Analytical Instruments GmbH, Kleve, Germany).

The adsorption capacity *q_ads_*, mass (mg) of adsorbed Dy^3+^, Tb^3+^, and Hg^2+^ ions per mass (g) of γFe_2_O_3_@SiO_2_–NH_2_ or CoFe_2_O_4_@SiO_2_–NH_2_ adsorbents and adsorption efficiency *q_ads_*_,%_ were calculated by the following equations:

(4) $$q_{ads}=\frac{\left(c_{ads,0\;}-c_{ads,e}\right)\;\cdot \;M_{ads\;}\cdot \;V}{m_{ads}}\;\;\left(\frac{mg}{g}\right)$$

(5) $$q_{ads,\%}=\frac{c_{ads,0}-c_{ads,e}}{c_{ads,0}}\;\left(\%\right)$$

where *c_ads_*_,0_ (mol/L) and *c_ads_*_,*e*_ (mol/L) relate to the initial and equilibrium concentrations of Dy^3+^, Tb^3+^, and Hg^2+^ ions, respectively, *V* (L) denotes the solution volume, *M_ads_* (g/mol) is the molar mass of adsorbate, and *m_ads_* (g) is the mass of adsorbent NPs.

Furthermore, desorption of adsorbed Dy^3+^, Tb^3+^, and Hg^2+^ ions from γFe_2_O_3_@SiO_2_–NH_2_ and CoFe_2_O_4_@SiO_2_–NH_2_ adsorbent surfaces was performed by mixing adsorbents with the prepared 1-M aqueous solution of HNO_3_ at 25 °C for 1 h. Desorption capacity was determined using the ICP-OES method and calculated by Equation (6):

(6) $$q_{des,\%}=\frac{c_{des}}{c_{ads}}\;\cdot 100\;\left(\%\right)$$

where *c_des_* (mg/g) is the concentration of adsorbate desorbed and *c_ads_* (mg/g) is the concentration of adsorbate adsorbed. Results for adsorption and desorption of Dy^3+^, Tb^3+^, and Hg^2+^ ions are shown in Table 1.

### 3.4. Toxicity Study of MNPs

#### 3.4.1. Cell Cultures

Four different types of healthy cell were used; human-skeletal-muscle-derived cells (SKMDCs), human fibroblasts, murine macrophage cells (RAW264.7), and human-umbilical-vein endothelial cells (HUVECs).

The SKMDCs were maintained in an F-10 nutrient medium supplemented with 25% fetal bovine serum (FBS), 1% penicillin/streptomycin (P/S), 0.1% insulin, 0.01% fibroblast-growth factor (FGF), and 0.01% epidermal growth factor (EGF). Fibroblasts were maintained in RPMI medium supplemented with 10% FBS and 1% P/S. The RAW264.7 cells were maintained in Dulbecco’s Modified Eagle’s Medium (DMEM) supplemented with 10% FBS and 1% P/S. The HUVECs were maintained in Endothelial cell Growth Medium 2 supplemented with FBS (2%), EGF (5 ng/mL), basic FGF (10 ng/mL), insulin-like growth factor (ILGF) (20 ng/mL), vascular endothelial growth factor (VEGF) (0.5 ng/mL), ascorbic acid (1 µg/mL), heparin (22.5 µg/mL), and hydrocortisone (0.2 µg/mL), 1% P/S. All cell types were allowed to grow in a humidified atmosphere at 37 °C under 5% CO_2_.

#### 3.4.2. Cytotoxicity Study

For cell-viability experiments, cells were seeded in a 96-well plate in 200 µL of their respective culture media; 24 h after cell growth, cells were treated with different concentrations of NPs and incubated for 3 days. Control cells were treated with the vehicle. After the incubation time, cells were incubated for 4 h with 0.5 mg mL^−1^ of 3-(4,5-dimethylthiazol-2-yl)-2,5-diphenyltetrazoliumbromide (MTT). After MTT incubation, MTT/medium was removed, and the precipitated violet crystals were dissolved in ethanol/DMSO (1:1, *v:v*) solution with shaking for 20 min. The absorbance was measured at 540 nm. The percentage (%) of live cells was calculated as Ab_test_/Ab_control_ 100. The experiment was performed three times.

#### 3.4.3. In Vitro Hemolytic Studies

Human-blood samples were obtained from a local blood bank (Établissement Français du Sang, Occitanie, France). Blood samples were collected in lithium heparin and stored at 4 °C until use. In total, 10 mL of blood were centrifuged at 1500 rpm for 5 min, after which the obtained platelet-poor plasma (PPP) was removed (~5 mL). The blood pellet was washed with 5 mL of phosphate-buffered saline (PBS) and mixed by inversion followed by centrifugation at 1500 rpm for 5 min, a process that was repeated 5 times.

The obtained red blood cells (RBCs) were diluted with PBS (1:10, *v*/*v*), and then treated with nanoparticles at concentrations ranging from 0 to 500 µg/mL, after which they were incubated at 37 °C for 1 h. The positive controls were RBCs treated with 1% Triton X-100, and the negative control was PBS (diluent). After incubation, samples were centrifuged at 1500 rpm for 5 min and the obtained supernatant was transferred to a polystyrene 96-well plate for reading at 540 nm, corresponding to the free hemoglobin band, using Thermo Scientific™ Multiskan SkyHigh Microplate Spectrophotometer. The hemolysis percentage was calculated as = (OD_test_ − OD_PBS_/OD_positive control_ − OD_PBS_) × 100, where OD is the optical density.

#### 3.4.4. Toxicity in Zebrafish Embryos

Fertilized wild-type AB zebrafish embryos were obtained from the laboratory facility of molecular mechanisms in neurodegenerative dementia (MMDN), Inserm U1198, Montpellier University, collected and maintained at 28 °C and 14-h-light/10-h-dark cycle.

At 7 h post-fertilization (hpf), embryos were examined under the microscope, and only embryos that developed normally were selected for the study. The 7 hpf embryos (15 per group) were placed in 12-well plates and exposed to 4 mL of water containing 0, 10, 50, 125, and 500 mg/L NPs. The exposure to NPs started at 7 hpf and ended at 96 hpf. The percentages of survival, mortality, and hatching of embryos were recorded using ZEISS Stemi 508 stereo microscope (ZEISS International, Oberkochen, Germany) at 24, 48, 52, 56, 72, and 96 hpf. The experiment was performed twice.

## 4. Conclusions

Superparamagnetic γFe_2_O_3_@SiO_2_–NH_2_ and CoFe_2_O_4_@SiO_2_–NH_2_ core@shell crystalline NPs were synthesized in a simple manner using two different approaches the classical coprecipitation method and the sol-gel technique, to obtain functionalized nano-sized superparamagnetic adsorbents designed for the binding and recycling of Dy^3+^, Tb^3+^, and Hg^2+^ ions from aqueous solutions. The synthesized spherical superparamagnetic γFe_2_O_3_@SiO_2_–NH_2_ and CoFe_2_O_4_@SiO_2_–NH_2_ NPs have excellent characteristics related to their structural, morphological, and surface properties, as well as their thermal stability, functionality, electrokinetic charge, and magnetic responsiveness. These properties were confirmed by TEM/HRTEM/EDXS, FT-IR, XRD, TGA, BET, DLS, and VSM.

Each individual material phase in the prepared γFe_2_O_3_@SiO_2_–NH_2_ and CoFe_2_O_4_@SiO_2_–NH_2_ adsorbent NPs plays a significant role in the adsorption processes. The superparamagnetic γFe_2_O_3_ and CoFe_2_O_4_ monodomain cores give the adsorbents the necessary magnetic properties and magnetic response in an external magnetic field, while the SiO_2_ amorphous shell allows the magnetic cores to be chemically and thermally stable and, due to the high content of hydroxyl groups on its surfaces, allows the high-density grafting of amino functional groups, which is necessary for interactions with metal ions. As Dy^3+^ and Tb^3+^ preferentially react with amino groups, unlike Hg^2+^, APTMS allows a higher capacity for their adsorption. The maximum adsorption capacities of Dy^3+^, Tb^3+^, and Hg^2+^ ions by the γFe_2_O_3_@SiO_2_–NH_2_ are 4.0 mg/g, 4.7 mg/g, and 2.1 mg/g, respectively, and 4.7 mg/g, 6.2 mg/g, and 1.2 mg/g by the CoFe_2_O_4_@SiO_2_–NH_2_. These values were obtained with a mass adsorbate of 50 mg, a contact time of 120 min, an initial concentration of Dy^3+^, Tb^3+^, and Hg^2+^ ions of 2 × 10^−6^ mol/L, and a temperature of 25 °C. The adsorption efficiency toward the Dy^3+^, Tb^3+^, and Hg^2+^ ions ranged from 83% to 98% for both the γFe_2_O_3_@SiO_2_–NH_2_ and the CoFe_2_O_4_@SiO_2_–NH_2_. In the post-adsorption treatment of the γFe_2_O_3_@SiO_2_–NH_2_ and CoFe_2_O_4_@SiO_2_–NH_2_ adsorbents in an acidic medium at pH 4.5, the Dy^3+^, Tb^3+^, and Hg^2+^ ions were completely desorbed from their surfaces. The desorption efficiency was 100%.

The toxicity assessment of the prepared adsorbents provided information on the relationship between their minimum dose and their responses to adverse effects on SKMDCs, fibroblasts, RAW264.7, and HUVECs, under the expected exposure conditions. The results showed the low toxicity of all the nanoparticles in the fibroblasts; however, higher toxicity was observed in macrophage RAW264.7 cells treated with γFe_2_O_3_@SiO_2_–NH_2_ and CoFe_2_O_4_@SiO_2_–NH_2_. The recording of the survival, mortality, and hatching percentages of zebrafish embryos exposed to different concentrations of the nanoparticles showed no toxicity compared to the control until 96 hpf, even at a high adsorbent concentration of 500 mg/L.

## Figures and Tables

**Table 1:** Results of the adsorption and desorption tests for Dy^3+^, Tb^3+^, and Hg^2+^ ions.

NPs	Adsorption Efficiency *q*_ads,%_ (%)			Adsorption Capacity *q*_ads_ (mg/g)			Desorption Efficiency *q*_des_ (%)		
	Dy^3+^	Tb^3+^	Hg^2+^	Dy^3+^	Tb^3+^	Hg^2+^	Dy^3+^	Tb^3+^	Hg^2+^
γ-Fe_2_O_3_@SiO_2_–NH_2_	83.1	89.3	94.3	4.0	4.7	2.1	100	100	100
CoFe_2_O_4_@SiO_2_–NH_2_	97.9	98.4	92.1	4.7	6.2	1.2	100	100	100

**Table 2:** The adsorption capacity of Dy^3+^, Tb^3+^, and Hg^2+^ with various adsorbent materials.

Adsorbent (NPs)	Adsorbate	Adsorption Conditions					Adsorption/Desorption Characteristics			Ref.
		*c*_ads,0_ (mg/L)	*γ*_ads,NPs_ (g/L)	*t*_ads_ (min)	*T*_ads_ (°C)	pH	*q*_ads_ (mg/g)	*q*_ads,%_ (%)	*q*_des,%_ (%)	
	**Dysprosium (Dy^3+^)**									
Fe_3_O_4_@SiO_2_@polyaniline–graphene oxide	Dy^3+^	0.01	0.4	2	25	4	16.0	98	95	[125]
Fe_3_O_4_–C_18_–chitosan–DETA	Dy^3+^	50	1.0	720	25	7	28.3	>80	>95	[126]
γ-Fe_2_O_3_-NH_4_OH@SiO_2_ (APTMS)	Dy^3+^	8.125	3.0	120	25	7	23.2	94	N/A	[84]
Synthetic-polymer-based magnetic adsorbent (M-PPTA)	Dy^3+^	50	3.0	130	25	5.5	24.0	98.4	>84	[127]
Polymeric adsorbents modified with ethylenediamine (EDA) and diglycolamic acid (DGA)	Dy^3+^	162.5	10.0	4320	25	1	18.4	30	N/A	[128]
Chemically activated carbons from spent-coffee waste	Dy^3+^	5.0	0.3	120	25	4	31.26	96	N/A	[129]
Physically activated carbons from spent-coffee waste							33.52	99		
*Ulva lactuca*—Chlorophyta (green)	Dy^3+^	0.5	3.0	4320	25	N/A ^(1)^	0.570	89	N/A	[130]
*Gracilaria* sp.—Rhodophyta (red)							0.526	84		
Fe^0^–SiO_2_–PA/SiO_2_–DTPA	Dy^3+^	1.5	0.5	30	21	3	1.85	N/A	N/A	[131]
γFe_2_O_3_@SiO_2_–NH_2_	Dy^3+^	32	2.5	120	25	4	4.0	83.1	100	This work
CoFe_2_O_4_@SiO_2_–NH_2_	Dy^3+^	32	2.5	120	25	4	4.7	97.9	100	This work
	**Terbium (Tb**^3+^)									
Fe_3_O_4_@SiO_2_@polyaniline-graphene oxide	Tb^3+^	0.01	0.4	2	25	4	11.8	98	95	[125]
Fe^0^–SiO_2_–PA/SiO_2_–DTPA	Tb^3+^	1.5	0.5	30	21	3	1.4	N/A	N/A	[131]
Molecular-sieve zeolite	Tb^3+^	20	0.5	2880	25	5	2.59	80	>60	[132]
*B. cereus* biomass-supported zeolite							5.07			
Multi-walled carbon nanotubes with tannic acid (TA-MWCNTs)	Tb^3+^	40	5	60	20	5	8.55	N/A	>95	[133]
γ-Fe_2_O_3_–NH_4_OH@SiO_2_ (APTMS)	Tb^3+^	0.32	1.5	120	25	7	0.204	93	N/A	[134]
γFe_2_O_3_@SiO_2_–NH_2_	Tb^3+^	32	2.5	120	25	4	4.7	89.3	100	This work
CoFe_2_O_4_@SiO_2_–NH_2_	Tb^3+^	32	2.5	120	25	4	6.2	98.4	100	This work
	**Mercury (Hg^2+^)**									
CoFe_2_O_4_–chitosan–graphene	Hg^2+^	20	0.12	230	50	7	361.0	90	<5	[135]
Polypyrrole-functionalized magnetic Kaolin (Ppy-Fe_3_O_4_/kaolin)	Hg^2+^	50	0.05	420	42	7.2	317.1	N/A	>90	[136]
CoFe_2_O_4_@SiO_2_–NH_2_	Hg^2+^	20	0.1	720	25	7	149.3	N/A	75	[137]
CoFe_2_O_4_@SiO_2_–EDTA	Hg^2+^	20	0.1	720	25	7	103.3	>90	>90	[138]
γ-Fe_2_O_3_@NH_2_	Hg^2+^	200	2.25	30	25	7	85.6	84	100	[122]
Fe_3_O_4_	Hg^2+^	100	2.5	720	23	N/A ^(2)^	28.0	<40	N/A	[139]
Fe_3_O_4_–Ag^0^							71.3	>80		
Rice-husk-activated carbon (RHAC)	Hg^2+^	20	0.2	60	25	5	55.87	N/A	N/A	[140]
Magnetic poly(vinyl alcohol)—procion blue MX-3G	Hg^2+^	400	5.0	10	20	6	69.2	>94	95	[141]
Magnetic poly(vinyl alcohol) (mPVAL)							0.57			
Amino-functionalized SiO_2_ particles (NH_2_@SiO_2_)	Hg^2+^	100	2.25	60	25	4	3.75	88	100	[123]
Activated carbon	Hg^2+^	0.1–300	2.3	1440	22	7.4	2.5	95	N/A	[142]
Gold-NP-coated silica							1.4	96		
γFe_2_O_3_@SiO_2_–NH_2_	Hg^2+^	40	2.5	120	25	4	2.1	94.3	100	This work
CoFe_2_O_4_@SiO_2_–NH_2_	Hg^2+^	40	2.5	120	25	4	1.2	92.1	100	This work

^(1)^ Seawater (salinity 0.175 mol/L–0.525 mol/L); ^(2)^ pH not adjusted.

## Data Availability

No such data that were created with the scope to share it publically.

## Supplementary Materials

The following supporting information can be downloaded at: https://www.mdpi.com/article/10.3390/ijms241210072/s1.
