# Supplementary material for: Superparamagnetic Spinel-Ferrite Nano-Adsorbents Adapted for Hg2+, Dy3+, Tb3+ Removal/Recycling: Synthesis, Characterization, and Assessment of Toxicity
Source: Int J Mol Sci. 2023 Jun 13;24(12):10072. doi: 10.3390/ijms241210072 (PMC10299025; doi:10.3390/ijms241210072)
Supplement: Supplementary file 1 [file ijms-24-10072-s001.zip › ijms-2355628-supplementary.pdf]

SUPPLEMENTARY FILE

Thermogravimetric analysis

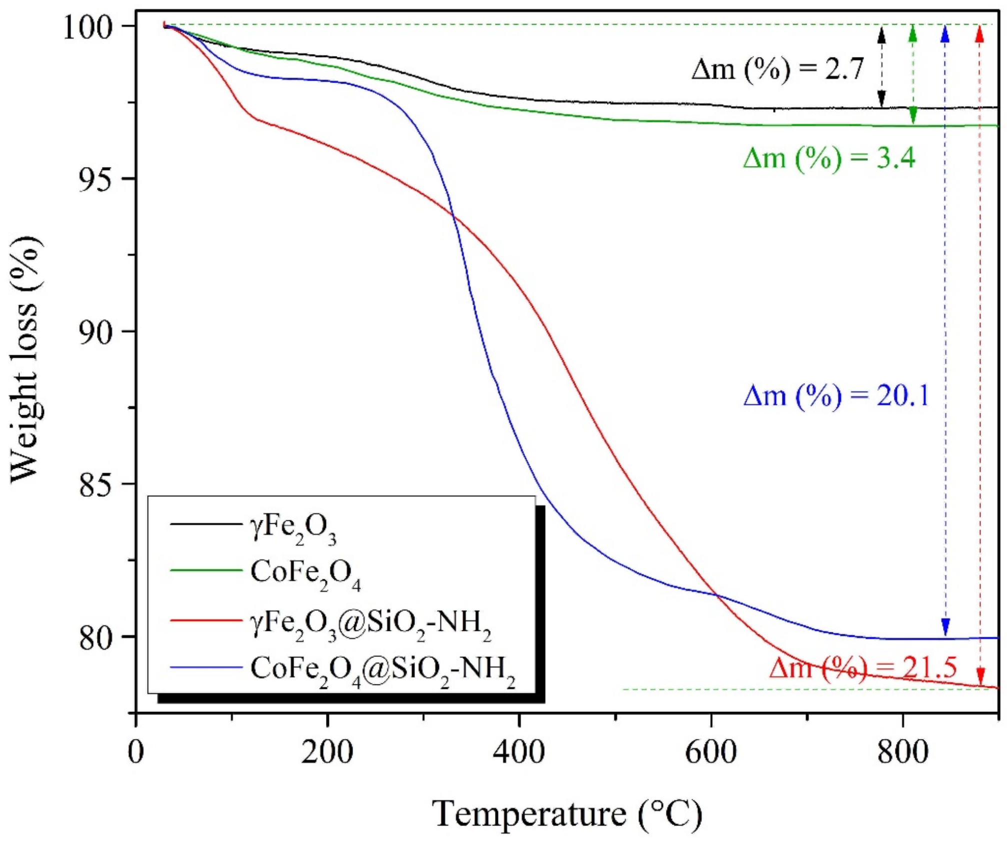

Figure S1. Thermogravimetric analysis of the prepared γFe_2O_3, $CoFe_2O_4$, $\gamma Fe_2O_3@SiO_2{-}NH_2$, and $CoFe_2O_4@SiO_2{-}NH_2$ NPs.

Potentiometric titration

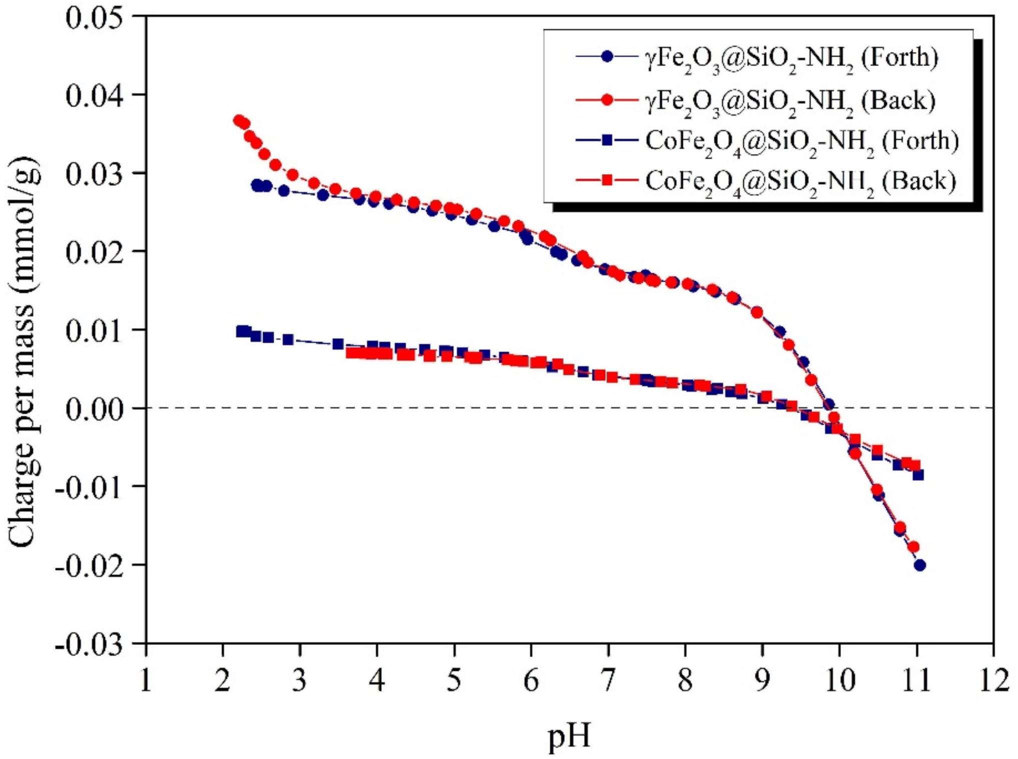

Figure S2. Potentiometric charge titration isotherms as a function of pH for $\gamma Fe_2O_3@SiO_2{-}NH_2$ and $CoFe_2O_4@SiO_2{-}NH_2$ NPs

Table S1. List of various adsorbent materials with their toxicological assessment in biological systems.

Assay	NM(s)	Model	Organ/tissue	Exposure	Dose range	Toxicity	Ref.
In vitro	Magnetic, SiO_2-coated	HEK293 (human)	Kidney	12 h	Up to 1.0 μg/μL	Toxic	[143]
In vitro	SiO_2	A549 (human)	Lung	24 h	0.1 to 6 $\mu g/cm^2$	Toxic	[144]
In vivo	MWCNT	Rat	Lung	6 h (aerosol) + 90-day post-exposure period	11 mg/m^3	Toxic	[145]
In vivo	TiO_2 (anatase) and hydroxylated fullerenes	Danio rerio (embryo)	Whole-organism Microinjection	48 h incubation	40 μg/mL fullerenes and 170 ng/mL	Toxic	[146]
In vitro	Ag	CaCo-2 (human)	Colon	24 h	2.5 and 25 μg/mL	Toxic	[147]
In vitro	Au, functionalised with anti-sense cDNAs	HCT-116 (human)	Colon	48 h	Equivalent to 30 nM of cDNAs	Toxic	[148]
In vitro	$\gamma Fe_2O_3@SiO_2-NH_2$	Human	Human skeletal muscles derived cells (SKMDCs), human fibroblasts, murine macrophage cells (RAW264.7), and human umbilical vein	4 h	0.5 mg mL^{-1} of 3-(4,5- dimethylthiazol-2-yl)-2,5-diphenyltetrazoliumbromide (MTT)	Non-toxic	This work

			endothelial cells (HUVECs)				
In vitro	$CoFe_2O_4@SiO_2-NH_2$	Human	Human skeletal muscles derived cells (SKMDCs), human fibroblasts, murine macrophage cells (RAW264.7), and human umbilical vein endothelial cells (HUVECs)	4 h	0.5 mg mL^{-1} of 3-(4,5-dimethylthiazol-2-yl)-2,5-diphenyltetrazoliumbromide (MTT)	Non-toxic	This work
In vivo	$\gamma Fe_2O_3@SiO_2-NH_2$	Zebrafish (Embryos)	Wild-type AB zebrafish embryos	7 h	0, 10, 50, 125, and 500 mg/L	Non-toxic	This work
In vivo	$CoFe_2O_4@SiO_2-NH_2$	Zebrafish (Embryos)	Wild-type AB zebrafish embryos	7 h	0, 10, 50, 125, and 500 mg/L	Non-toxic	This work

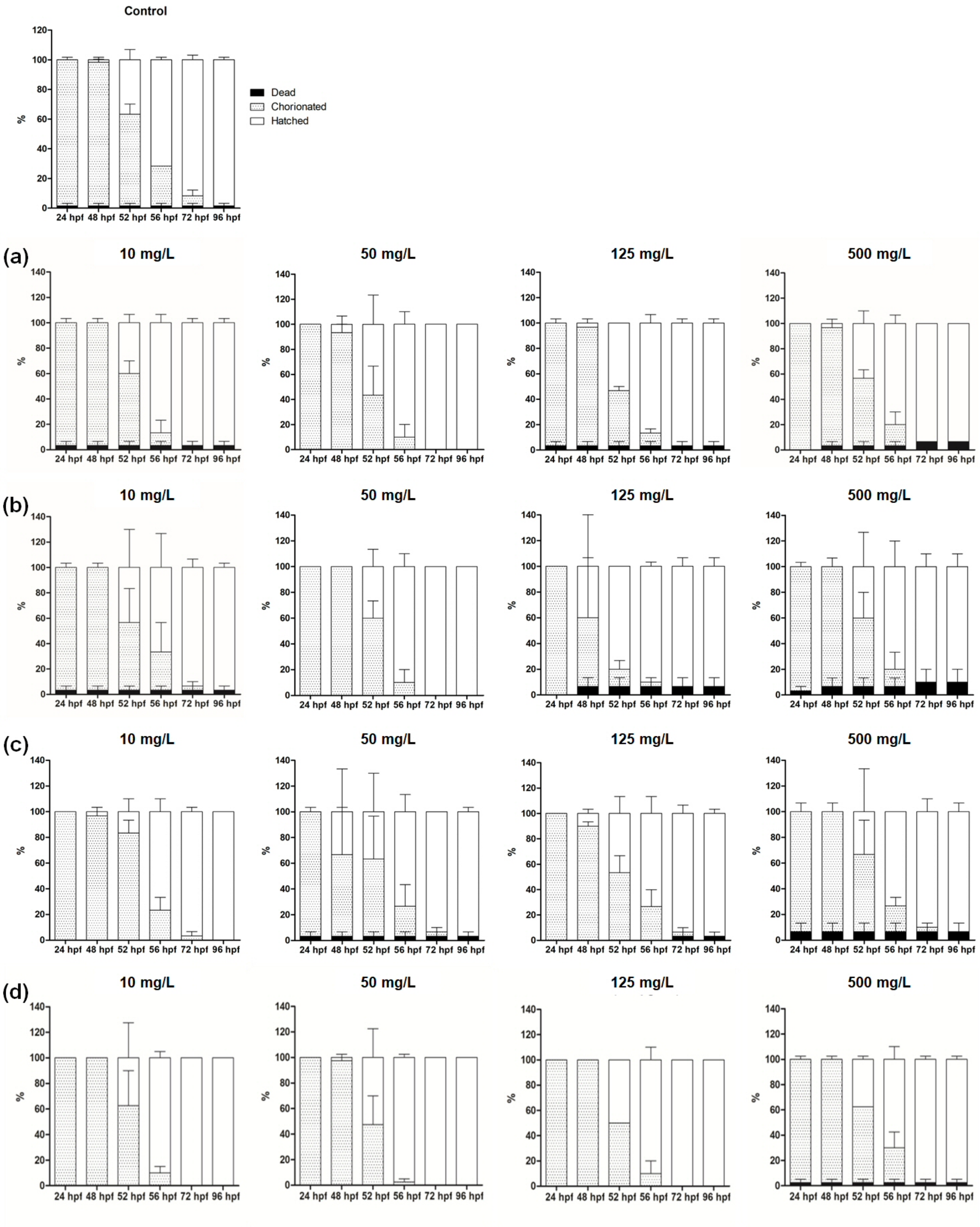

Figure S3. Zebrafish embryo development expressed as percentages of dead, chorionated, and hatched, in water containing different concentrations of nanoparticles (a) γFe_2O_3 (b) $\gamma Fe_2O_3@SiO_2{-}NH_2$ c) $CoFe_2O_4$, and d)

$CoFe_2O_4@SiO_2-NH_2$ for 24, 48, 52, 56, 72, and 96 hours post fertilization (hpf). Data are presented as mean ± SEM of two independent experiments.
